# Nutritional care in rehabilitation and acute care of stroke patients: a systematic review of clinical practice guidelines

**DOI:** 10.3389/fstro.2025.1558019

**Published:** 2025-04-10

**Authors:** Karina Siewers, Katrine Svaerke, Amira Eliza Rosenørn, Hanne Christensen

**Affiliations:** Department of Neurology, Bispebjerg Hospital, Copenhagen, Denmark

**Keywords:** stroke, nutrition, systematic review, guidelines and recommendations, rehabilitation

## Abstract

**Background:**

Malnutrition and nutritional care are significant challenges for healthcare professionals treating stroke patients, in both acute care and during rehabilitation. This study aimed to assess and synthesize the nutritional care recommendations in clinical practice guidelines (CPGs) for managing malnutrition risk in stroke patients, evaluate the supporting evidence, identify research gaps, and assess the quality of the CPGs.

**Methods:**

Three databases, along with National Medical Association websites and nutrition journals, were searched for CPGs published between 2019 and 2024 that provided recommendations on nutritional care for stroke patients. Two independent reviewers performed data extraction, and three reviewers independently assessed CPG quality and clinical applicability (using AGREE II and AGREE-REX tools).

**Results:**

13 CPGs were included in this review. These were of varying quality, with overall moderate AGREE II total scores [mean (SD), 55.2% (21.8%)]. Only two CPGs had an overall quality score above 70% based on AGREE-REX total scores, while five were categorized as poor quality (scores < 40%). Most guidelines strongly recommended early dysphagia screening upon hospital admission, including the provision of texture-modified foods for patients with dysphagia. However, recommendations on malnutrition, nutritional support, and supplementation were often either absent or inconsistent across guidelines and recommendations were mostly based on moderate to weak evidence.

**Conclusion:**

This study highlights the critical need for more rigorous research, standardized approaches, and patient-centered studies to improve and optimize nutritional care practices for stroke patients.

**Systematic review registration:**

https://www.crd.york.ac.uk/PROSPERO/view/CRD42024498430, PROSPERO registration ID: CRD42024498430.

## Introduction

Malnutrition poses a significant challenge amongst patients with stroke, with up to 49% developing malnutrition post-stroke with further worsening during hospitalization and rehabilitation (Mosselman et al., 2013; Foley et al., 2009). Dysphagia increases the risk of malnutrition in post-stroke patients; however, malnutrition can occur in patients regardless of the presence of dysphagia (Foley et al., 2009). Managing malnutrition is complex and involves health care professionals from various professions.

## Background

Healthcare providers require access to high-quality Clinical Practice Guidelines (CPGs). CPGs influence day-to-day clinical decisions and serve as a crucial tool in standardizing health care practices, bridging the gap between clinical practice and evidence-based scientific support.

Given the complexity of and diversity in nutritional interventions and assessment for stroke management, CPGs are needed to guide clinical decision-making, elevate patient care, and optimize outcomes. The study objectives were to assess the quality of eligible CPGs on nutritional care for stroke patients and to identify and synthesize key recommendations from the included CPGs.

## Methods

The systematic review of CPGs was registered at PROSPERO (ID CRD42024498430) in advance and conducted in accordance with the PRISMA 2020 checklist for systematic reviews (see Supplementary material S1) (Page et al., 2021). Eligibility criteria were created in adherence to the PICAR statements [Population(s) and Clinical Area(s), Intervention(s), Comparator(s), Attributes of CPGs and Recommendation characteristics] framework (see Table 1) (Johnston et al., 2019).

**Table 1:** PICAR statement for inclusion and exclusion of studies.

**Parameter**	**Inclusion criteria**	**Exclusion criteria**
Population and clinical area(s)	- Human adults (≥18 years) - Admitted to hospital or rehabilitation facility after ischemic stroke or spontaneous hemorrhagic stroke - Clinical indications: • Swallowing issues • Malnutrition • Rehabilitation	Guidelines that do not specify stroke care
Intervention(s)	Any nutritional interventions including, but not limited to tube feeding, texture modification, dietary modification, gastrostomy feeding, parenteral/enteral nutrition oral supplements, fortified foods Screening for malnutrition, dietary recommendations	Nutritional care outside hospital and rehabilitation facilities.
Comparator(s)	No comparator	No comparator
**A**ttributes of CPGs	- **Language:** English language - **Year:** Published 2019–2024 - **Publishing region:** All available - **Version:** Only the latest version of CPGs is of interest - **Development process:** CPGs are explicitly evidence-based^†^ - **System of rating evidence:** CPGs use a system to rate the level of evidence behind recommendations (e.g., GRADE) - **Scope:** CPGs primarily focused on the management of stroke and stroke rehabilitation - **Recommendations:** CPGs will only be included if they report one or more eligible recommendations of interest	
**R**ecommendation characteristics and other considerations	**Duration of treatment**: Recommendations on duration of treatment with nutritional care or dietary supplementation are of particular interest **Level of confidence:** Each recommendation must be accompanied by an explicit level of confidence **Interventions:** Recommendations must explicitly discuss ≥ 1 intervention of interest **Comparators:** Recommendations are not required to compare an intervention of interest to another. **Locating recommendations:** Within CPG text, tables and/or algorithms	

^†^CPGs must show evidence that a literature search was performed.

CPG, clinical practice guideline; GRADE, grading of recommendations, assessment, development, and evaluations.

### Data sources and search strategy

On Jan 5th 2024, one investigator (KSi) searched MEDLINE and Embase using a predefined search strategy. We also carried out a search of the NICE database, additional supplementary searches for CPGs on National Medical Association webpages, health organizations webpages. Guidelines published since 2019 were included. Search results were limited to those published in English. Full details of the search strategy can be found in the Supplementary material S2.

### Selection of guidelines and recommendations

**Figure 1:**
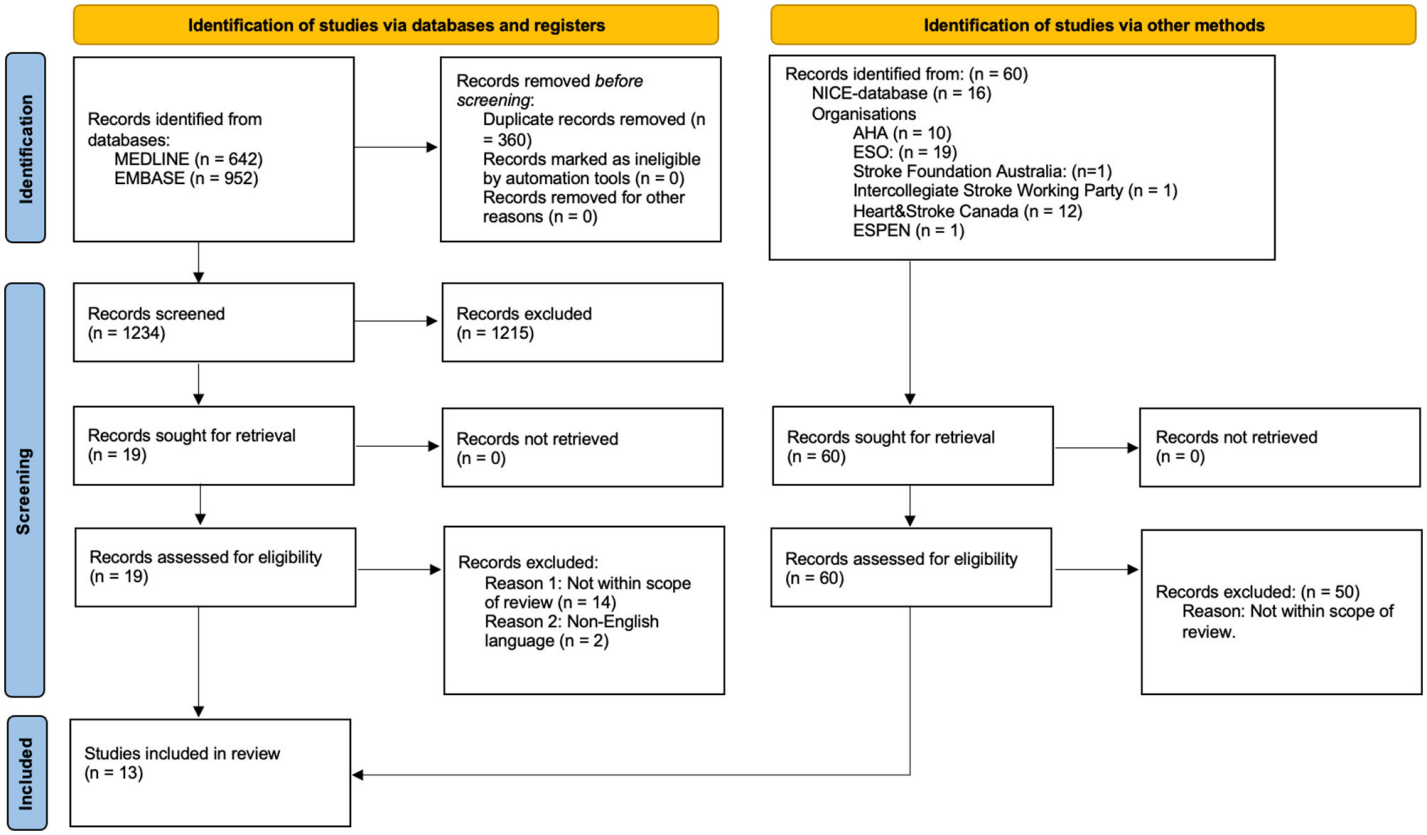
PRISMA 2020 flow diagram for new systematic reviews which included searches of databases, registers and other sources.

The records that returned from MEDLINE and Embase were imported to the web-based software platform (Covidence systematic review software: www.covidence.org). Two reviewers (KSi and HC) independently reviewed titles and abstracts of all records returned from the literature search. In case of uncertainty, consensus was reached by discussion. Next, full texts of the remaining records were obtained and examined for inclusion, based on predefined eligibility criteria outlined in the PICAR statement (Table 1). CPGs not meeting the inclusion criteria were excluded, explanations are illustrated in the PRISMA flowchart (Figure 1).

### Data extraction

Two investigators (KSi + KSv) independently screened each recommendation reported in the included CPGs for eligibility against the PICAR statement. If the recommendation was deemed eligible, the data was extracted from each study and entered into a pre-defined data extraction form. The extraction sheet was compiled using Microsoft Excel spreadsheet, any discrepancies were solved by the fourth reviewer HC. Recommendations from each guideline were grouped into four categories: Dysphagia, Malnutrition Screening, Nutritional Supplementation and Tube-feeding.

### Quality assessment

The Appraisal of Guidelines for Research and Evaluation II (AGREE II) tool (Brouwers et al., 2010) was used to assess the included guidelines. AGREE II was developed to provide a systematic framework for assessing the quality of CPGs. All included guidelines were independently appraised by three reviewers (KSi, AR, and KSv). Differences in scores of ≥3 were discussed, to avoid the risk of individual misinterpretations of the guidelines. For overall quality assessment, mean domain scores were categorized as “high-quality” if the domain score was >70%, “moderate-quality” when 40–70% and “poor-quality” when the domain score was < 40%, as recommended by AGREE II. With regard to these CPGs, the following criteria were considered: if four of the six domains obtained a score of ≥60%, the CPG was recommended.

### Clinical applicability

To ensure appraisal of guideline recommendations the Appraisal of Guidelines Research and Evaluation—Recommendations Excellence (AGREE-REX) tool was used (Brouwers et al., 2020). The AGREE-REX tool is a valid tool to assess guideline recommendations. A consensus approach was used to reach agreement with the AGREE-REX item scores. Recommendations were considered as “high-quality” if the domain score was >70%, “moderate-quality” when 40–70% and “poor-quality” when the domain score was < 40%.

## Results

Our search strategy revealed 1,653 potential records. After removal of duplicates and title and abstract screening 78 records remained for full-text review and eligibility assessment. Studies were most often excluded for not being clinical practice guidelines. Twelve CPGs were identified at included. One additional CPG (Thibault et al., 2021) was not identified during the initial screening process through the EMBASE or Medline search but was later found through a gray literature search after data extraction and subsequently included in the study. Therefore, 13 clinical practice guidelines were included in the data synthesis (Figure 1; Supplementary material S3).

### Quality assessment

Guidelines were heterogeneous in quality with AGREE II total scores of overall moderate qualities [mean (SD), 55.2% (21.8%)]. Generally individual quality domain scores for domain 5 (Applicability) [mean (SD), 35.6% (33.0%)] were of poor-quality, with insufficient reporting on resource implications of application, barriers to its application and presentation of monitoring. Domain 4 (Clarity of Presentation) scored highest across guidelines ranging from 41–98% (moderate- to high quality) (see Supplementary material S4, S5). Four guidelines (Stroke Foundation, 2024; NICE, 2023; Heran et al., 2024; Teasell et al., 2020) were rated high-quality with overall domain scores > 70%, whereas three (Dziewas et al., 2021a; Minelli et al., 2022a,b) guidelines presented poor-quality with overall domain score < 40%. According to predefined standards, seven CPGs achieved acceptable quality and were recommended (Stroke Foundation, 2024; NICE, 2023; Heran et al., 2024; Teasell et al., 2020; Dziewas et al., 2021a; National Clinical Guideline for Stroke, 2023; NICE, 2022), while six were not recommended for use in clinical practice (Thibault et al., 2021; Dziewas et al., 2021a; Minelli et al., 2022a,b; Powers et al., 2019; Greenberg et al., 2022).

### Clinical applicability

Two of the CPG recommendations had an overall quality score above 70% (Stroke Foundation, 2024; NICE, 2023), thus being classified as high-quality. Six CPG recommendations (Heran et al., 2024; Teasell et al., 2020; Dziewas et al., 2021a; National Clinical Guideline for Stroke, 2023; NICE, 2022; Greenberg et al., 2022) had an overall quality score classified as moderate-quality (40–70%) thus resulting in five CPG recommendations (Thibault et al., 2021; Heran et al., 2024; Dziewas et al., 2021a; Minelli et al., 2022a; Powers et al., 2019) categorized as poor-quality. The quality scores for domain 1 (Clinical Applicability) and 3 (Implementability) ranged from 30–94% and 19–94%, respectively, revealing big heterogeneity between CPG recommendations. Domain 2 (Values and Preferences) scored generally lower with a mean score of 30.2%, ranging between 1–74%. Especially presenting insufficient information on values and preferences of policy/decision makers and guideline developers. For individual scoring of AGREE-REX for each CPG see Supplementary material S6.

### Synthesis of recommendations from the eligible CPGs: variations across guidelines

#### Dysphagia

Six of the eligible CPGs (Stroke Foundation, 2024; Heran et al., 2024; Dziewas et al., 2021a; National Clinical Guideline for Stroke, 2023; NICE, 2022; Powers et al., 2019) provide recommendations on the timing of dysphagia screening; however, their guidance varies. Some specify that screening should be conducted *as soon as possible* (Heran et al., 2024; Dziewas et al., 2021a), others recommend it *before patients receive any oral food or fluids* (Stroke Foundation, 2024; Powers et al., 2019), while some recommend that it be *conducted by a specialist assessment within a timeframe of 24 to 72 hours* (National Clinical Guideline for Stroke, 2023; NICE, 2022). Six of the included CPGs (Thibault et al., 2021; Stroke Foundation, 2024; NICE, 2023; Dziewas et al., 2021a,b; National Clinical Guideline for Stroke, 2023) recommended texture modifications for patients with dysphagia. Two CPGs (Teasell et al., 2020; Minelli et al., 2022a) recommended involvement of dietitians for patients with dysphagia needing alterations in food texture, and two CPGs (Dziewas et al., 2021a,b) recommended additional monitoring of fluid balance, nutritional intake, and risk of complications (e.g., pneumonia and dehydration).

#### Screening for malnutrition

Six CPGs (Thibault et al., 2021; Stroke Foundation, 2024; Teasell et al., 2020; Minelli et al., 2022a; National Clinical Guideline for Stroke, 2023; NICE, 2022) provided recommendations on the timing on the timing of malnutrition screening. All six recommend screening upon admission, while four of them (Thibault et al., 2021; Stroke Foundation, 2024; National Clinical Guideline for Stroke, 2023; NICE, 2022) additionally advise conducting weekly follow-up screenings thereafter. None of the CPGs specify a particular screening tool to be used.

#### Nutritional intervention

Seven CPGs (Thibault et al., 2021; Stroke Foundation, 2024; Minelli et al., 2022a; Dziewas et al., 2021a; National Clinical Guideline for Stroke, 2023; NICE, 2022; Powers et al., 2019) provide recommendations on nutritional supplementations; however, their guidance varies. All seven CPGs recommend providing nutritional supplementation for patients at risk of malnutrition, while five of them (Thibault et al., 2021; Stroke Foundation, 2024; Minelli et al., 2022a; Dziewas et al., 2021a; Powers et al., 2019) specifically state that nutritional supplementation should also be given to patients who are already malnourished, in addition to those at risk. The provision of nutritional support was elaborated further in four CPGs (Thibault et al., 2021; Heran et al., 2024; National Clinical Guideline for Stroke, 2023; NICE, 2022). I.S.C. (National Clinical Guideline for Stroke, 2023) and T.I.A. (NICE, 2022) suggested oral supplement, dietary advice and/or tube feeding for malnourished patients. C.S.M. (Heran et al., 2024) suggested the development of an individualized management plan addressing dysphagia therapy. Six CPGs (Thibault et al., 2021; Stroke Foundation, 2024; Minelli et al., 2022a; Dziewas et al., 2021a; National Clinical Guideline for Stroke, 2023; NICE, 2022) recommended the avoidance of nutritional supplements for stroke patients who are adequately nourished.

#### Enteral feeding

Six CPGs address the timing of enteral feeding for patients with post-stroke dysphagia and insufficient oral intake, but their recommendations vary. One CPG suggest starting nasogastric tube feeding (Dziewas et al., 2021a), two recommend initiation within 24 hours of admission (National Clinical Guideline for Stroke, 2023; NICE, 2022), another two within 3 days (Heran et al., 2024; Minelli et al., 2022a), and one advises nasogastrc tube starting within the first 7 days (Powers et al., 2019).

Regarding gastrostomy (PEG) or gastric-jejunal (G-J) tubes, six CPGs (Heran et al., 2024; Teasell et al., 2020; Minelli et al., 2022a; National Clinical Guideline for Stroke, 2023; NICE, 2022; Powers et al., 2019) offer differing recommendations. Three specify timing for replacing the nasogastric tube. One CPG (Powers et al., 2019) recommend PEG if swallowing impairment exceeds 2–3 weeks, another (National Clinical Guideline for Stroke, 2023) suggests it after 4 weeks or if the nasogastric tube is not tolerated, and a third (Minelli et al., 2022a) advises it if enteral nutrition is needed for over 3 weeks. Two CPGs (Heran et al., 2024; Teasell et al., 2020) do not define exact timing but recommend G-J tubes for prolonged enteral feeding, while one CPG suggest PEG if the patients are unable to tolerate nasogastric tube.

## Discussion

This systematic review examined 13 CPGs focusing on nutritional care for stroke patients in both acute and rehabilitation settings. Most guidelines strongly recommended early dysphagia screening upon admission to hospital, including the provision of texture modified food for patients with dysphagia. However, recommendations on nutritional supplementation varied between guidelines and were based on moderate to weak evidence.

### Quality of guidelines

The scope of the guidelines varied, encompassing the comprehensive management of all stroke types, focusing specifically on either acute or rehabilitation care post-stroke, and addressing either ischemic or hemorrhagic stroke subtypes exclusively. Most guidelines were focused on dysphagia assessment and management, possibly due to the larger body of research evidence on post-stroke dysphagia compared to other areas of nutritional assessment and interventions in post-stroke care. Recommendations regarding provision of nutritional supplementations were ambiguous and nonspecific and were sparsely covered by guidelines. Guidelines rarely discussed the treatment goals for dysphagic and malnourished patients, but emphasized the importance of dysphagia screening to minimize the risk of pneumonia and similar complications.

Generally, individual quality domain scores for domain 5 (Applicability) in AGREE II were poor with insufficient reporting on resource implications, barriers to application, and monitoring. This aligns with findings from other systematic reviews on CPGs (Jolliffe et al., 2018; O'Donnell et al., 2020; Montero-Odasso et al., 2021), indicating that the applicability domain often scores the lowest across various healthcare topics. The CPGs failed to adequately identify and describe potential facilitators, barriers, and cost implications of their recommendations. Challenges in implementing recommendations should be better addressed in future clinical practice guidelines to improve their clinical applicability.

#### Dysphagia

The number of randomized controlled trials (RCT) for dysphagia screening in stroke patients has seen only a marginal increase in the last 5 years. A recent review and meta-analysis (Sherman et al., 2021) identified five RCTs for dysphagia screening post-stroke. To broaden the perspective the meta-analysis included 22 observational studies; however, these 27 studies variously compared four different screening tools including “the Acute Screening of Swallowing in Stroke/TIA” and “the Gugging Swallowing Screen (GUSS)”, making it difficult to draw definite conclusions on optimal screening tools. The included studies also looked at different screening thresholds of 24 h, 4 h and 79 min after admission, which agrees with the disagreements between included CPGs. However, there is broad agreement throughout studies, that dysphagia screening of adult stroke patients reduces their risk of pneumonia, mortality and length of stay (Sherman et al., 2021; Bray, 2017), A Delphi-based consensus study of experts in Turkey (Umay et al., 2021) from 2021 was excluded as it did not meet the predefined inclusion criteria. However, its recommendations largely align with those presented in the included CPGs, with the exception of lacking guidance on the timing of dysphagia screening. This alignment suggests that clinical experts managing stroke patients with dysphagia broadly support the approaches outlined in existing CPGs, reinforcing their relevance in clinical practice.

A recent study from 2024 aimed at improving compliance with best practice recommendations for dysphagia screening in stroke patients (Shen et al., 2024) found that such screenings are rarely performed according to guidelines. The study identified key barriers, including a lack of knowledge and the absence of a standardized approach to guide screening. Notably, after implementing targeted training sessions and developing a structured protocol, compliance rates rose significantly to 97.9%. This highlights a crucial point: while best practice guidelines provide essential direction, effective implementation requires a department-wide strategy that ensures adequate training, knowledge dissemination, and clear protocols to support sustained adherence.

#### Screening for malnutrition

Six CPGs (Thibault et al., 2021; Stroke Foundation, 2024; Teasell et al., 2020; Minelli et al., 2022a; National Clinical Guideline for Stroke, 2023; NICE, 2022) had strong recommendations on malnutrition screening upon admission to hospital, interestingly all of which were based on guideline development consensus, underlining the lack of evidence behind screening tools and the benefits of early screening. No specific methods or timing for assessing malnutrition were mentioned, despite the availability of numerous screening tools. A recent literature review (Di Vincenzo et al., 2023) evaluated studies on the assessment of nutritional risk in stroke patients and highlighted the need for future research to identify the most appropriate assessment tools.

#### Nutritional intervention

Six CPGs (Thibault et al., 2021; Stroke Foundation, 2024; Minelli et al., 2022a; Dziewas et al., 2021a; National Clinical Guideline for Stroke, 2023; NICE, 2022) recommended the avoidance of nutritional supplements for stroke patients who are adequately nourished. The guideline recommendations are based on the 2005 FOOD trial (Dennis et al., 2005a), which investigated oral nutritional supplementation in 4,023 non-dysphagic stroke patients. The trial found a non-significant 0.7% reduction in the risk of death, and did not support the use of routine oral supplementation after stroke. The study faced several methodological limitations, including inconsistent and informal nutritional assessments, with 63% of patients evaluated solely by bedside observation. Nutritional intake and status were not closely monitored, leaving uncertainty about whether the supplemented group actually received more calories or protein than the control group. This is particularly important as previous studies (Milne et al., 2009) have suggested a reduction in normal dietary intake in patients receiving oral supplements. Furthermore, 28% of patients discontinued supplementation prematurely, and compliance was not comprehensively assessed. Although the trial demonstrated no benefit of nutritional supplementation for non-dysphagic, adequately nourished stroke patients, malnutrition remains prevalent among stroke patients and worsens outcomes, including mortality, infection rates, and functional recovery. A later study (Rabadi et al., 2008) addressed some of these limitations by providing intensive nutritional supplementation to malnourished stroke patients (defined as ≥2.5% unintentional weight loss within 2 weeks post-stroke), including both dysphagic and non-dysphagic patients. This study found significantly improved outcomes in the treated group, contradicting the FOOD trial's conclusions. Unfortunately, these findings have not influenced current CPG recommendations on nutritional supplementation. Further research is needed to determine optimal nutritional strategies for stroke patients. Further research is necessary to determine the optimal nutritional strategies for stroke patients.

#### Enteral feeding

There is disagreement among guidelines about the timing of enteral feeding after stroke, ranging from 24 h to 7 days. Interestingly all CPGs reference the FOOD II trial (Dennis et al., 2005b) as the main evidence. The trial tested early vs. delayed feeding and concluded that nasogastric feeding should begin within the first few days of hospital admission, which agrees with the CPGs suggesting initiation within 3 days. However, the inclusion criteria allowed early feeding to be defined as up to 10 days after hospital admission, which conflicts with the guidelines' recommendation of 24 hours to 7 days, as well as the trial's conclusions. The trial provides no data on median time from admission to initiation of enteral feeding. While the trial showed a non-significant reduction in the risk of death with early feeding, this evidence does not fully support the strict timing recommended by the guidelines and future studies are needed to conclude on the optimal timing of enteral feeding.

### Strength and limitations

An established methodology was followed adhering to PRISMA guidelines and utilizing the AGREE II and AGREE-REX tools to assess the quality of the guidelines and their recommendations. The AGREE tools are relatively novel (most recent updates: 2017 and 2019), but the study only included CPGs from 2019, allowing all included CPGs to adhere to AGREE development standards. The exclusion of non-English CPGs may limit the overall generalizability of the findings to other settings, such as nursery homes and rehabilitation facilities outside hospitals. The trial only included publicly available online guidelines, which may have excluded smaller, local guidelines that are not easily accessible online. This systematic review on nutritional care omitted recommendations on oral hygiene, despite evidence indicating that proper oral hygiene can alleviate eating difficulties and support adequate nutritional intake (Cardoso, 2023).

### Implications for future research

Stroke care is complex and rely on expert clinicians and healthcare professionals administering the best and most rigorous treatments to ensure optimal outcomes. Despite great strides in stroke care, malnutrition remains a significant challenge, with up to 49% of stroke patients becoming malnourished, possibly worsening rehabilitation potential and physical outcomes. This is an area overlooked in CPGs, which lack specific details on the implementation of recommendations, such as malnutrition screening tools and the exact timing and type of nutritional interventions. Future research should aim to identify, compare and validate specific malnutrition screening tools tailored for stroke patients to ensure uniformity across clinical settings. Given the wide range of nutritional supplements available, comparing trials is challenging. A large-scale platform trial that systematically evaluates scientifically developed; promising supplements would be valuable for future research. Non-dysphagic stroke patients remain at risk of malnutrition despite being able to consume regular foods. Investigating the underlying causes—such as nausea, anxiety, depression, or loss of appetite post-stroke—would be highly valuable. Gaining insight into these factors could help inform the design of future intervention studies aimed at addressing this issue effectively.

## Conclusion

The systematic review highlights the critical role of nutritional care in the management of stroke patients in both acute and rehabilitation settings. Despite the heterogeneity in quality and the definitions of recommendations, most CPGs emphasize the importance of early dysphagia screening, regular malnutrition assessment, and appropriate texture modification or enteral feeding tailored to patient needs. Only 75% of CPGs presented recommendations on management of malnutrition and nutritional supplementations, in which weak and moderate recommendations were made, underlining the imperative need for more rigorous studies and more standardized approaches and patient centered research to optimize nutritional care practices. Future CPGs should better address the clinical applicability of their CPGs, with more explicit considerations on the barriers to implementation, strategies to its uptake and resource implications of applying the guideline and recommendations should address values and preferences of patients, policy makers and target users.

## Data Availability

The original contributions presented in the study are included in the article/Supplementary material, further inquiries can be directed to the corresponding author.
