# Supplementary material for: Nutritional care in rehabilitation and acute care of stroke patients: a systematic review of clinical practice guidelines
Source: Front Stroke. 2025 Apr 10;4:1558019. doi: 10.3389/fstro.2025.1558019 (PMC12802607; doi:10.3389/fstro.2025.1558019)
Supplement: Supplementary file 2 [file Table_2.docx]

**Supplementary Material Table S2: Search Strategy**

**MEDLINE:**

1. ((("stroke*" OR "post-stroke" OR "poststroke" OR "apoplectic" OR "apoplex*" OR "intracranial haemorrhage" OR "intracerebral haemorrhage" OR "cerebral haemorrhage" OR "basal ganglia haemorrhage" OR "cerebral intraventricular haemorrhage" OR "brain haemorrhage" OR "intracranial hemorrhage" OR "intracerebral hemorrhage" OR "cerebral hemorrhage" OR "basal ganglia hemorrhage" OR "cerebral intraventricular hemorrhage" OR "brain hemorrhage" OR "brain bleed" OR "cerebrovascular accident" OR "intracranial embolism" OR "intracranial thrombosis" OR "Cerebrovascular accident" OR "brain infarction" OR "brain ischemia" OR "intracranial ischemia" OR "intracranial infarction" OR "intracerebral ischemia" OR "intracerebral infarction" OR "cerebral infarction" OR "cerebral ischemia" OR "brain ischemia" OR "transient ischemic attack" OR "cerebral artery infarction" OR "cerebral artery occlusion")
2. AND ("nutrition*" OR "nutrient*" OR "nutritional*" OR "nourishment" OR "fortified food*" OR "enriched meal*" OR "fortified meal*" OR "enriched food*" OR "food therapy" OR "micronutrient*" OR "macronutrient*" OR "vitamin*" OR "dietary supplement*" OR "herbal supplement*" OR "mineral supplement*" OR "oral supplement*" OR "probiotic*" OR "prebiotic*" OR "tube feeding" OR "feeding tube*" OR "PEG-tube" OR "PEG tube" OR "Percutaneous endoscopic gastrostomy" OR "parenteral feeding" OR "enteral feeding" OR "intravenous feeding" OR "IV feeding" OR "nasogastric feeding" OR "nutrient-fortified" OR "specialized diet*" OR "specialised diet*" OR "therapeutic diet*" OR "modified diet*" OR "diet modification*" OR "tailored diet*")
3. AND ("guideline*"[Title/Abstract] OR "consensus"[Title/Abstract] OR "best practice"[Title/Abstract] OR "recommendation*"[Title/Abstract])
4. AND (“national*"[Title/Abstract] OR “international*"[Title/Abstract] OR “regional*"[Title/Abstract] OR “global*"[Title/Abstract]
5. Only English language
6. Jan 1^st^ 2019 - Jan 5^th^ 2024

**Embase:**

1. ((("stroke*" OR "post-stroke" OR "poststroke" OR "apoplectic" OR "apoplex*" OR "intracranial haemorrhage" OR "intracerebral haemorrhage" OR "cerebral haemorrhage" OR "basal ganglia haemorrhage" OR "cerebral intraventricular haemorrhage" OR "brain haemorrhage" OR "intracranial hemorrhage" OR "intracerebral hemorrhage" OR "cerebral hemorrhage" OR "basal ganglia hemorrhage" OR "cerebral intraventricular hemorrhage" OR "brain hemorrhage" OR "brain bleed" OR "cerebrovascular accident" OR "intracranial embolism" OR "intracranial thrombosis" OR "Cerebrovascular accident" OR "brain infarction" OR "brain ischemia" OR "intracranial ischemia" OR "intracranial infarction" OR "intracerebral ischemia" OR "intracerebral infarction" OR "cerebral infarction" OR "cerebral ischemia" OR "brain ischemia" OR "transient ischemic attack" OR "cerebral artery infarction" OR "cerebral artery occlusion")
2. AND ("nutrition*" OR "nutrient*" OR "nutritional*" OR "nourishment" OR "fortified food*" OR "enriched meal*" OR "fortified meal*" OR "enriched food*" OR "food therapy" OR "micronutrient*" OR "macronutrient*" OR "vitamin*" OR "dietary supplement*" OR "herbal supplement*" OR "mineral supplement*" OR "oral supplement*" OR "probiotic*" OR "prebiotic*" OR "tube feeding" OR "feeding tube*" OR "PEG-tube" OR "PEG tube" OR "Percutaneous endoscopic gastrostomy" OR "parenteral feeding" OR "enteral feeding" OR "intravenous feeding" OR "IV feeding" OR "nasogastric feeding" OR "nutrient-fortified" OR "specialized diet*" OR "specialised diet*" OR "therapeutic diet*" OR "modified diet*" OR "diet modification*" OR "tailored diet*")
3. AND ("guideline*" OR "consensus"OR "best practice" OR "recommendation*")
4. AND (“national*"[Title/Abstract] OR “international*"[Title/Abstract] OR “regional*"[Title/Abstract] OR “global*"[Title/Abstract]
5. Only English language
6. Jan 1^st^ 2019 – Jan 5^th^ 2024

**NICE Database Webpage:**

<https://www.nice.org.uk/guidance>

NICE > NICE Guidance > Conditions and diseases > Neurological conditions

**European Stroke Organization Webpage:**

<https://eso-stroke.org/guidelines/eso-guideline-directory/>

“Acute Stroke” and “Rehabilitation and long-term consequences of stroke”

**Stroke Foundation Australia:**

<https://strokefoundation.org.au/what-we-do/for-health-professionals/clinical-guidelines>

**Intercollegiate Stroke Working Party (UK and Ireland):**

<https://www.strokeguideline.org/>

**American Stroke Association:**

<https://professional.heart.org/en/guidelines-and-statements/guidelines-and-statements-search>

1. Search: “Stroke”

2. Filter: Jan 1^st^ 2019 – Jan 5^th^ 2024

3. Document type: “Clinical Practice Guidelines”

**Heart&Stroke Canada:**

<https://www.strokebestpractices.ca/recommendations>

**European Society for Clinical Nutrition and Metabolism (ESPEN):**

<https://www.espen.org/guidelines/espen-scientific-guidelines-pdf-versions>
