# Supplementary material for: Nutritional care in rehabilitation and acute care of stroke patients: a systematic review of clinical practice guidelines
Source: Front Stroke. 2025 Apr 10;4:1558019. doi: 10.3389/fstro.2025.1558019 (PMC12802607; doi:10.3389/fstro.2025.1558019)
Supplement: Supplementary file 3 [file Table_3.docx]

| **Supplementary Material Table S3: Characteristics of included clinical practice guidelines** | | | | | | |
| --- | --- | --- | --- | --- | --- | --- |
| **CPG abbreviation** | **Countries** | **Organization** | **Publication Date** | **Multidisciplinary Team** | **Target Patients** | **Grading System** |
| A.I.S.^18^ | USA | AHA/ASA | Oct 2019 | 19 members of the writing group, representing various areas of medical expertise. | Adult patients with acute arterial ischemic stroke. | ACC/AHA: class (strength) of recommendation (COR) |
| I.C.H.^19^ | USA | AHA/ASA | May 2022 | Vascular neurologists,  neurocritical care specialists, neurological surgeons,  an emergency physician, a haematologist, a rehabilitation  medicine physician, a board-certified acute care nurse  practitioner, a fellow-in-training, and a lay/patient representative. | Spontaneous intracerebral hemorrhage in adult patients. | ACC/AHA: class (strength) of recommendation (COR) |
| E.S.O.^15^ | Europe | ESO | Oct 2021 | Three neurologists, four speech-and-language therapists, a phoniatrician, a surgeon, a geriatrician, a gastro-enterologist, a stroke physician, a pharmacist, a guideline methodologist and a rehabilitation physician. | Patients with post-stroke dysphagia. | GRADE for RCT and SIGN for NRCT summarized into: grades of quality evidence. high, moderate, low and very low |
| S.F.M.^8^ | Australia and New Zealand | Stroke Foundation | Jul 2023 | Multidisciplinary team including 119 personnel from various professions including physicians, nurses, psychologists, dietitians, speech therapists, physiotherapists and occupational therapists. | Stroke and transient ischemic attack in adults. | GRADE for RCT and SIGN for NRCT summarized into: grades of quality evidence. high, moderate, low and very low |
| I.S.C.^16^ | UK and Ireland | Intercollegiate Stroke Working Party | May 2023 | Multidisciplinary team including physicians, language and speech therapists, physiotherapists, dentists, neuropsychologists, occupational therapists, radiologists and patient representatives. | Adults who have a stroke regardless of age, gender, type of stroke and location. | SIGN |
| S.R.A.^9^ | NICE | United Kingdom | Oct 2023 | Multidisciplinary team including 21 individuals including physicians, speech and language therapist, lay members, neuropsychologists, nurses, physiotherapists, occupational therapists, dietitians and an orthoptist. | Stroke in adults and young people aged 16 and over | GRADE - grading of recommendations, assessment, development and evaluation |
| T.I.A.^17^ | NICE | United Kingdom | Apr 2022 | Multidisciplinary team including 15 individuals including physicians, a paramedic, radiographer, a nurse, two lay members, a radiologist, a physiotherapist and a neurosurgeon. | People aged over 16 who have had a stroke or TIA. | GRADE - grading of recommendations, assessment, development and evaluation |
| N.G.D.^12^ | German Society of Neurology | Germany | May 2021 | Experts from 27 medical societies. | Not disclosed. | Not disclosed |
| B.R.1.^13^ | Brazilian Academy of Neurology | Brazil | Aug 2022 | 31 experts of undisclosed professions. | Stroke patients. | Class I, II(a/b) and III |
| B.R.2.^14^ | Brazilian Academy of Neurology | Brazil | Nov 2022 | 35 experts of undisclosed professions. | Stroke patients. | Class I, II(a/b) and III |
| C.S.M.^10^ | Heart&Stroke | Canada | Dec 2022 | Medical doctors, nurses, social workers and paramedics. | People with current or very recent symptoms of acute stroke or transient ischemic attack. | GRADE - grading of recommendations, assessment, development and evaluation |
| C.R.R.^11^ | Heart&Stroke | Canada | Jan 2020 | Medical doctors, psychologists, nurses, dieticians. | People of all stroke types, including acute ischemic stroke, transient ischemic attack, intracerebral hemorrhage and subarachnoid hemorrhage. | GRADE - grading of recommendations, assessment, development and evaluation |
| E.S.P.^7^ | European Society of Nutrition and Metabolism | Europe | Sep 2021 | Six physicians and five dietitians. | Patients in hospitals, rehabilitation centers and nursing homes. | SIGN |
| Abbreviations: CPG: clinical practice guideline, AHA: American heart association, ASA, American stroke association, ACC: American college of cardiology, COR: class of recommendation, GRADE: grading of recommendation, assessment, development and evaluation, RCT: randomized controlled trial, SIGN: Scottish intercollegiate guidelines network, NRCT: non-randomized controlled trial, NICE: national institute of health and care excellence. | | | | | | |
