# Supplementary material for: Nutritional care in rehabilitation and acute care of stroke patients: a systematic review of clinical practice guidelines
Source: Front Stroke. 2025 Apr 10;4:1558019. doi: 10.3389/fstro.2025.1558019 (PMC12802607; doi:10.3389/fstro.2025.1558019)
Supplement: Supplementary file 4 [file Table_4.docx]

**Supplementary Material Table S4: AGREE-II Assessment for all studies**

| **Scaled domain score** | **Domain 1** | **Domain 2** | **Domain 3** | **Domain 4** | **Domain 5** | **Domain 6** | **Total** |
| --- | --- | --- | --- | --- | --- | --- | --- |
| A.I.S.^1^ | 50 | 37 | 56 | 67 | 1 | 47 | 44 |
| I.C.H.^2^ | 63 | 48 | 58 | 80 | 10 | 86 | 54 |
| E.S.O.^3^ | 85 | 65 | 74 | 85 | 21 | 94 | 68 |
| S.F.M.^4^ | 100 | 87 | 88 | 98 | 68 | 72 | 86 |
| I.S.C.^5^ | 80 | 80 | 58 | 57 | 60 | 92 | 67 |
| S.R.A.^6^ | 96 | 89 | 81 | 93 | 83 | 50 | 83 |
| T.I.A.^7^ | 81 | 83 | 25 | 80 | 61 | 22 | 53 |
| N.G.D.^8^ | 44 | 4 | 20 | 65 | 7 | 61 | 28 |
| B.R.1.^9^ | 54 | 35 | 12 | 52 | 4 | 11 | 24 |
| B.R.2.^10^ | 39 | 24 | 12 | 41 | 0 | 14 | 19 |
| C.S.M.^11^ | 59 | 76 | 63 | 80 | 71 | 94 | 71 |
| C.R.R.^12^ | 61 | 89 | 56 | 85 | 71 | 94 | 71 |
| E.S.P.^13^ | 80 | 44 | 60 | 61 | 6 | 33 | 49 |
| Mean [SD] | 68.6 [19.7] | 58.5 [28.3] | 51.0 [25.6] | 72.6 [16.9] | 35.6 [33.0] | 59.2 [32.0] | 55.2 [21.8] |

**A.I.S**^1^

Domain 1 (Scope and Purpose)

|  | Item 1 | Item 2 | Item 3 | Total |
| --- | --- | --- | --- | --- |
| Appraiser 1 (KSi) | 4 | 3 | 6 | 13 |
| Appraiser 2 (KSv) | 3 | 3 | 5 | 11 |
| Appraiser 3 (AR) | 6 | 1 | 5 | 12 |
| Total | 13 | 7 | 16 | 36 |
| Maximum possible score = 7 (strongly agree) x 3 (items) x 3 (appraisers) = 63  Minimum possible score = 1 (strongly disagree) x 3 (items) x 3 (appraisers) = 9 | | | | |
| $\frac{36-9}{63-9}*100=50 \%$ | | | | |

Domain 2 (Stakeholder Involvement)

|  | Item 4 | Item 5 | Item 6 | Total |
| --- | --- | --- | --- | --- |
| Appraiser 1 (KSi) | 3 | 1 | 6 | 10 |
| Appraiser 2 (KSv) | 3 | 2 | 5 | 10 |
| Appraiser 3 (AR) | 2 | 1 | 6 | 9 |
| Total | 8 | 4 | 17 | 29 |
| Maximum possible score = 7 (strongly agree) x 3 (items) x 3 (appraisers) = 63  Minimum possible score = 1 (strongly disagree) x 3 (items) x 3 (appraisers) = 9 | | | | |
| $\frac{29-9}{63-9}*100=37 \%$ | | | | |

Domain 3 (Rigour of Development)

|  | Item 7 | Item 8 | Item 9 | Item 10 | Item 11 | Item 12 | Item 13 | Item 14 | Total |
| --- | --- | --- | --- | --- | --- | --- | --- | --- | --- |
| Appraiser 1 (KSi) | 6 | 4 | 5 | 5 | 5 | 5 | 3 | 3 | 36 |
| Appraiser 2 (KSv) | 5 | 3 | 4 | 4 | 5 | 3 | 3 | 3 | 30 |
| Appraiser 3 (AR) | 7 | 2 | 7 | 4 | 5 | 7 | 4 | 2 | 38 |
| Total | 18 | 9 | 16 | 13 | 15 | 15 | 10 | 8 | 104 |
| Maximum possible score = 7 (strongly agree) x 8 (items) x 3 (appraisers) = 168  Minimum possible score = 1 (strongly disagree) x 8 (items) x 3 (appraisers) = 24 | | | | | | | | | |
| $\frac{104-24}{168-24}*100=56 \%$ | | | | | | | | | |

Domain 4 (Clarity of Presentation)

|  | Item 15 | Item 16 | Item 17 | Total |
| --- | --- | --- | --- | --- |
| Appraiser 1 (KSi) | 5 | 3 | 7 | 15 |
| Appraiser 2 (KSv) | 5 | 2 | 7 | 14 |
| Appraiser 3 (AR) | 5 | 5 | 6 | 16 |
| Total | 15 | 10 | 20 | 45 |
| Maximum possible score = 7 (strongly agree) x 3 (items) x 3 (appraisers) = 63  Minimum possible score = 1 (strongly disagree) x 3 (items) x 3 (appraisers) = 9 | | | | |
| $\frac{45-9}{63-9}*100=67\%$ | | | | |

Domain 5 (Applicability)

|  | Item 18 | Item 19 | Item 20 | Item 21 | Total |
| --- | --- | --- | --- | --- | --- |
| Appraiser 1 (KSi) | 1 | 1 | 1 | 1 | 4 |
| Appraiser 2 (KSv) | 1 | 1 | 1 | 1 | 4 |
| Appraiser 3 (AR) | 1 | 2 | 1 | 1 | 5 |
| Total | 3 | 4 | 3 | 3 | 13 |
| Maximum possible score = 7 (strongly agree) x 4 (items) x 3 (appraisers) = 84  Minimum possible score = 1 (strongly disagree) x 4 (items) x 3 (appraisers) = 12 | | | | | |
| $\frac{13-12}{84-12}*100=1 \%$ | | | | | |

Domain 6 (Editorial Independence)

|  | Item 22 | Item 23 | Total |
| --- | --- | --- | --- |
| Appraiser 1 (KSi) | 1 | 7 | 8 |
| Appraiser 2 (KSv) | 1 | 7 | 8 |
| Appraiser 3 (AR) | 1 | 6 | 7 |
| Total | 3 | 20 | 23 |
| Maximum possible score = 7 (strongly agree) x 2 (items) x 3 (appraisers) = 42  Minimum possible score = 1 (strongly disagree) x 2 (items) x 3 (appraisers) = 6 | | | |
| $\frac{23-6}{42-6}*100=47 \%$ | | | |

**I.C.H.**^2^

Domain 1 (Scope and Purpose)

|  | Item 1 | Item 2 | Item 3 | Total |
| --- | --- | --- | --- | --- |
| Appraiser 1 (KSi) | 5 | 4 | 6 | 15 |
| Appraiser 2 (KSv) | 4 | 4 | 6 | 14 |
| Appraiser 3 (AR) | 5 | 3 | 6 | 14 |
| Total | 14 | 11 | 18 | 43 |
| Maximum possible score = 7 (strongly agree) x 3 (items) x 3 (appraisers) = 63  Minimum possible score = 1 (strongly disagree) x 3 (items) x 3 (appraisers) = 9 | | | | |
| $\frac{43-9}{63-9}*100=63 \%$ | | | | |

Domain 2 (Stakeholder Involvement)

|  | Item 4 | Item 5 | Item 6 | Total |
| --- | --- | --- | --- | --- |
| Appraiser 1 (KSi) | 5 | 3 | 4 | 12 |
| Appraiser 2 (KSv) | 6 | 2 | 3 | 11 |
| Appraiser 3 (AR) | 6 | 3 | 3 | 12 |
| Total | 17 | 8 | 10 | 35 |
| Maximum possible score = 7 (strongly agree) x 3 (items) x 3 (appraisers) = 63  Minimum possible score = 1 (strongly disagree) x 3 (items) x 3 (appraisers) = 9 | | | | |
| $\frac{35-9}{63-9}*100=48 \%$ | | | | |

Domain 3 (Rigour of Development)

|  | Item 7 | Item 8 | Item 9 | Item 10 | Item 11 | Item 12 | Item 13 | Item 14 | Total |
| --- | --- | --- | --- | --- | --- | --- | --- | --- | --- |
| Appraiser 1 (KSi) | 5 | 4 | 4 | 6 | 6 | 6 | 4 | 1 | 36 |
| Appraiser 2 (KSv) | 4 | 2 | 4 | 5 | 6 | 6 | 3 | 1 | 31 |
| Appraiser 3 (AR) | 6 | 6 | 6 | 4 | 6 | 7 | 5 | 1 | 41 |
| Total | 15 | 12 | 14 | 15 | 18 | 19 | 12 | 3 | 108 |
| Maximum possible score = 7 (strongly agree) x 8 (items) x 3 (appraisers) = 168  Minimum possible score = 1 (strongly disagree) x 8 (items) x 3 (appraisers) = 24 | | | | | | | | | |
| $\frac{108-24}{168-24}*100=58 \%$ | | | | | | | | | |

Domain 4 (Clarity of Presentation)

|  | Item 15 | Item 16 | Item 17 | Total |
| --- | --- | --- | --- | --- |
| Appraiser 1 (KSi) | 7 | 5 | 7 | 19 |
| Appraiser 2 (KSv) | 7 | 4 | 7 | 18 |
| Appraiser 3 (AR) | 6 | 3 | 6 | 15 |
| Total | 20 | 12 | 20 | 52 |
| Maximum possible score = 7 (strongly agree) x 3 (items) x 3 (appraisers) = 63  Minimum possible score = 1 (strongly disagree) x 3 (items) x 3 (appraisers) = 9 | | | | |
| $\frac{52-9}{63-9}*100=80 \%$ | | | | |

Domain 5 (Applicability)

|  | Item 18 | Item 19 | Item 20 | Item 21 | Total |
| --- | --- | --- | --- | --- | --- |
| Appraiser 1 (KSi) | 1 | 3 | 1 | 2 | 7 |
| Appraiser 2 (KSv) | 1 | 2 | 1 | 1 | 5 |
| Appraiser 3 (AR) | 2 | 3 | 1 | 1 | 7 |
| Total | 4 | 8 | 3 | 4 | 19 |
| Maximum possible score = 7 (strongly agree) x 4 (items) x 3 (appraisers) = 84  Minimum possible score = 1 (strongly disagree) x 4 (items) x 3 (appraisers) = 12 | | | | | |
| $\frac{19-12}{84-12}*100=10 \%$ | | | | | |

Domain 6 (Editorial Independence)

|  | Item 22 | Item 23 | Total |
| --- | --- | --- | --- |
| Appraiser 1 (KSi) | 5 | 7 | 12 |
| Appraiser 2 (KSv) | 4 | 7 | 11 |
| Appraiser 3 (AR) | 7 | 7 | 14 |
| Total | 16 | 21 | 37 |
| Maximum possible score = 7 (strongly agree) x 2 (items) x 3 (appraisers) = 42  Minimum possible score = 1 (strongly disagree) x 2 (items) x 3 (appraisers) = 6 | | | |
| $\frac{37-6}{42-6}*100=86 \%$ | | | |

**E.S.O.**^3^

Domain 1 (Scope and Purpose)

|  | Item 1 | Item 2 | Item 3 | Total |
| --- | --- | --- | --- | --- |
| Appraiser 1 (KSi) | 7 | 7 | 5 | 19 |
| Appraiser 2 (KSv) | 7 | 6 | 6 | 19 |
| Appraiser 3 (AR) | 5 | 7 | 5 | 17 |
| Total | 19 | 20 | 16 | 55 |
| Maximum possible score = 7 (strongly agree) x 3 (items) x 3 (appraisers) = 63  Minimum possible score = 1 (strongly disagree) x 3 (items) x 3 (appraisers) = 9 | | | | |
| $\frac{55-9}{63-9}*100=85 \%$ | | | | |

Domain 2 (Stakeholder Involvement)

|  | Item 4 | Item 5 | Item 6 | Total |
| --- | --- | --- | --- | --- |
| Appraiser 1 (KSi) | 6 | 3 | 6 | 15 |
| Appraiser 2 (KSv) | 5 | 2 | 7 | 14 |
| Appraiser 3 (AR) | 7 | 2 | 6 | 15 |
| Total | 18 | 7 | 19 | 44 |
| Maximum possible score = 7 (strongly agree) x 3 (items) x 3 (appraisers) = 63  Minimum possible score = 1 (strongly disagree) x 3 (items) x 3 (appraisers) = 9 | | | | |
| $\frac{44-9}{63-9}*100=65 \%$ | | | | |

Domain 3 (Rigour of Development)

|  | Item 7 | Item 8 | Item 9 | Item 10 | Item 11 | Item 12 | Item 13 | Item 14 | Total |
| --- | --- | --- | --- | --- | --- | --- | --- | --- | --- |
| Appraiser 1 (KSi) | 7 | 5 | 7 | 2 | 6 | 7 | 3 | 7 | 44 |
| Appraiser 2 (KSv) | 7 | 3 | 7 | 1 | 7 | 7 | 2 | 7 | 41 |
| Appraiser 3 (AR) | 6 | 7 | 5 | 4 | 7 | 7 | 4 | 5 | 45 |
| Total | 20 | 15 | 19 | 7 | 20 | 21 | 9 | 19 | 130 |
| Maximum possible score = 7 (strongly agree) x 8 (items) x 3 (appraisers) = 168  Minimum possible score = 1 (strongly disagree) x 8 (items) x 3 (appraisers) = 24 | | | | | | | | | |
| $\frac{130-24}{168-24}*100=74 \%$ | | | | | | | | | |

Domain 4 (Clarity of Presentation)

|  | Item 15 | Item 16 | Item 17 | Total |
| --- | --- | --- | --- | --- |
| Appraiser 1 (KSi) | 6 | 6 | 6 | 18 |
| Appraiser 2 (KSv) | 7 | 7 | 7 | 21 |
| Appraiser 3 (AR) | 7 | 5 | 4 | 16 |
| Total | 20 | 18 | 17 | 55 |
| Maximum possible score = 7 (strongly agree) x 3 (items) x 3 (appraisers) = 63  Minimum possible score = 1 (strongly disagree) x 3 (items) x 3 (appraisers) = 9 | | | | |
| $\frac{55-9}{63-9}*100=85 \%$ | | | | |

Domain 5 (Applicability)

|  | Item 18 | Item 19 | Item 20 | Item 21 | Total |
| --- | --- | --- | --- | --- | --- |
| Appraiser 1 (KSi) | 4 | 3 | 1 | 2 | 10 |
| Appraiser 2 (KSv) | 3 | 2 | 1 | 1 | 7 |
| Appraiser 3 (AR) | 3 | 5 | 1 | 1 | 10 |
| Total | 10 | 10 | 3 | 4 | 27 |
| Maximum possible score = 7 (strongly agree) x 4 (items) x 3 (appraisers) = 84  Minimum possible score = 1 (strongly disagree) x 4 (items) x 3 (appraisers) = 12 | | | | | |
| $\frac{27-12}{84-12}*100=21 \%$ | | | | | |

Domain 6 (Editorial Independence)

|  | Item 22 | Item 23 | Total |
| --- | --- | --- | --- |
| Appraiser 1 (KSi) | 7 | 7 | 14 |
| Appraiser 2 (KSv) | 7 | 7 | 14 |
| Appraiser 3 (AR) | 7 | 5 | 12 |
| Total | 21 | 19 | 40 |
| Maximum possible score = 7 (strongly agree) x 2 (items) x 3 (appraisers) = 42  Minimum possible score = 1 (strongly disagree) x 2 (items) x 3 (appraisers) = 6 | | | |
| $\frac{40-6}{42-6}*100=94 \%$ | | | |

**S.F.M.**^4^

Domain 1 (Scope and Purpose)

|  | Item 1 | Item 2 | Item 3 | Total |
| --- | --- | --- | --- | --- |
| Appraiser 1 (KSi) | 7 | 7 | 7 | 21 |
| Appraiser 2 (KSv) | 7 | 7 | 7 | 21 |
| Appraiser 3 (AR) | 7 | 7 | 7 | 21 |
| Total | 21 | 21 | 21 | 63 |
| Maximum possible score = 7 (strongly agree) x 3 (items) x 3 (appraisers) = 63  Minimum possible score = 1 (strongly disagree) x 3 (items) x 3 (appraisers) = 9 | | | | |
| $\frac{63-9}{63-9}*100=100 \%$ | | | | |

Domain 2 (Stakeholder Involvement)

|  | Item 4 | Item 5 | Item 6 | Total |
| --- | --- | --- | --- | --- |
| Appraiser 1 (KSi) | 6 | 7 | 7 | 20 |
| Appraiser 2 (KSv) | 6 | 5 | 7 | 18 |
| Appraiser 3 (AR) | 5 | 7 | 6 | 18 |
| Total | 17 | 19 | 20 | 56 |
| Maximum possible score = 7 (strongly agree) x 3 (items) x 3 (appraisers) = 63  Minimum possible score = 1 (strongly disagree) x 3 (items) x 3 (appraisers) = 9 | | | | |
| $\frac{56-9}{63-9}*100=87 \%$ | | | | |

Domain 3 (Rigour of Development)

|  | Item 7 | Item 8 | Item 9 | Item 10 | Item 11 | Item 12 | Item 13 | Item 14 | Total |
| --- | --- | --- | --- | --- | --- | --- | --- | --- | --- |
| Appraiser 1 (KSi) | 4 | 7 | 7 | 5 | 7 | 7 | 6 | 7 | 50 |
| Appraiser 2 (KSv) | 7 | 7 | 7 | 4 | 7 | 7 | 6 | 7 | 52 |
| Appraiser 3 (AR) | 5 | 5 | 7 | 5 | 7 | 7 | 6 | 7 | 49 |
| Total | 16 | 19 | 21 | 14 | 21 | 21 | 18 | 21 | 151 |
| Maximum possible score = 7 (strongly agree) x 8 (items) x 3 (appraisers) = 168  Minimum possible score = 1 (strongly disagree) x 8 (items) x 3 (appraisers) = 24 | | | | | | | | | |
| $\frac{151-24}{168-24}*100=88 \%$ | | | | | | | | | |

Domain 4 (Clarity of Presentation)

|  | Item 15 | Item 16 | Item 17 | Total |
| --- | --- | --- | --- | --- |
| Appraiser 1 (KSi) | 6 | 7 | 7 | 20 |
| Appraiser 2 (KSv) | 7 | 7 | 7 | 21 |
| Appraiser 3 (AR) | 7 | 7 | 7 | 21 |
| Total | 20 | 21 | 21 | 62 |
| Maximum possible score = 7 (strongly agree) x 3 (items) x 3 (appraisers) = 63  Minimum possible score = 1 (strongly disagree) x 3 (items) x 3 (appraisers) = 9 | | | | |
| $\frac{62-9}{63-9}*100=98 \%$ | | | | |

Domain 5 (Applicability)

|  | Item 18 | Item 19 | Item 20 | Item 21 | Total |
| --- | --- | --- | --- | --- | --- |
| Appraiser 1 (KSi) | 5 | 7 | 7 | 1 | 20 |
| Appraiser 2 (KSv) | 6 | 6 | 7 | 2 | 21 |
| Appraiser 3 (AR) | 6 | 6 | 7 | 1 | 20 |
| Total | 17 | 19 | 21 | 4 | 61 |
| Maximum possible score = 7 (strongly agree) x 4 (items) x 3 (appraisers) = 84  Minimum possible score = 1 (strongly disagree) x 4 (items) x 3 (appraisers) = 12 | | | | | |
| $\frac{61-12}{84-12}*100=68 \%$ | | | | | |

Domain 6 (Editorial Independence)

|  | Item 22 | Item 23 | Total |
| --- | --- | --- | --- |
| Appraiser 1 (KSi) | 7 | 3 | 10 |
| Appraiser 2 (KSv) | 7 | 4 | 11 |
| Appraiser 3 (AR) | 7 | 4 | 11 |
| Total | 21 | 11 | 32 |
| Maximum possible score = 7 (strongly agree) x 2 (items) x 3 (appraisers) = 42  Minimum possible score = 1 (strongly disagree) x 2 (items) x 3 (appraisers) = 6 | | | |
| $\frac{32-6}{42-6}*100=72 \%$ | | | |

**I.S.C.**^5^

Domain 1 (Scope and Purpose)

|  | Item 1 | Item 2 | Item 3 | Total |
| --- | --- | --- | --- | --- |
| Appraiser 1 (KSi) | 7 | 5 | 7 | 19 |
| Appraiser 2 (KSv) | 7 | 4 | 7 | 18 |
| Appraiser 3 (AR) | 5 | 4 | 6 | 15 |
| Total | 19 | 13 | 20 | 52 |
| Maximum possible score = 7 (strongly agree) x 3 (items) x 3 (appraisers) = 63  Minimum possible score = 1 (strongly disagree) x 3 (items) x 3 (appraisers) = 9 | | | | |
| $\frac{52-9}{63-9}*100=80 \%$ | | | | |

Domain 2 (Stakeholder Involvement)

|  | Item 4 | Item 5 | Item 6 | Total |
| --- | --- | --- | --- | --- |
| Appraiser 1 (KSi) | 6 | 5 | 7 | 18 |
| Appraiser 2 (KSv) | 5 | 4 | 7 | 16 |
| Appraiser 3 (AR) | 7 | 5 | 6 | 18 |
| Total | 18 | 14 | 20 | 52 |
| Maximum possible score = 7 (strongly agree) x 3 (items) x 3 (appraisers) = 63  Minimum possible score = 1 (strongly disagree) x 3 (items) x 3 (appraisers) = 9 | | | | |
| $\frac{52-9}{63-9}*100=80 \%$ | | | | |

Domain 3 (Rigour of Development)

|  | Item 7 | Item 8 | Item 9 | Item 10 | Item 11 | Item 12 | Item 13 | Item 14 | Total |
| --- | --- | --- | --- | --- | --- | --- | --- | --- | --- |
| Appraiser 1 (KSi) | 7 | 6 | 4 | 6 | 5 | 5 | 4 | 1 | 38 |
| Appraiser 2 (KSv) | 7 | 5 | 3 | 5 | 4 | 4 | 2 | 1 | 31 |
| Appraiser 3 (AR) | 7 | 7 | 3 | 6 | 6 | 4 | 5 | 1 | 39 |
| Total | 21 | 18 | 10 | 17 | 15 | 13 | 11 | 3 | 108 |
| Maximum possible score = 7 (strongly agree) x 8 (items) x 3 (appraisers) = 168  Minimum possible score = 1 (strongly disagree) x 8 (items) x 3 (appraisers) = 24 | | | | | | | | | |
| $\frac{108-24}{168-24}*100=58 \%$ | | | | | | | | | |

Domain 4 (Clarity of Presentation)

|  | Item 15 | Item 16 | Item 17 | Total |
| --- | --- | --- | --- | --- |
| Appraiser 1 (KSi) | 5 | 4 | 5 | 14 |
| Appraiser 2 (KSv) | 4 | 3 | 4 | 11 |
| Appraiser 3 (AR) | 5 | 5 | 5 | 15 |
| Total | 14 | 12 | 14 | 40 |
| Maximum possible score = 7 (strongly agree) x 4 (items) x 3 (appraisers) = 84  Minimum possible score = 1 (strongly disagree) x 4 (items) x 3 (appraisers) = 12 | | | | |
| $\frac{40-12}{84-12}*100=57 \%$ | | | | |

Domain 5 (Applicability)

|  | Item 18 | Item 19 | Item 20 | Item 21 | Total |
| --- | --- | --- | --- | --- | --- |
| Appraiser 1 (KSi) | 6 | 7 | 6 | 1 | 20 |
| Appraiser 2 (KSv) | 7 | 6 | 4 | 1 | 18 |
| Appraiser 3 (AR) | 4 | 6 | 6 | 1 | 17 |
| Total | 17 | 19 | 16 | 3 | 55 |
| Maximum possible score = 7 (strongly agree) x 4 (items) x 3 (appraisers) = 84  Minimum possible score = 1 (strongly disagree) x 4 (items) x 3 (appraisers) = 12 | | | | | |
| $\frac{55-12}{84-12}*100=60 \%$ | | | | | |

Domain 6 (Editorial Independence)

|  | Item 22 | Item 23 | Total |
| --- | --- | --- | --- |
| Appraiser 1 (KSi) | 7 | 6 | 13 |
| Appraiser 2 (KSv) | 7 | 6 | 13 |
| Appraiser 3 (AR) | 7 | 6 | 13 |
| Total | 21 | 18 | 39 |
| Maximum possible score = 7 (strongly agree) x 2 (items) x 3 (appraisers) = 42  Minimum possible score = 1 (strongly disagree) x 2 (items) x 3 (appraisers) = 6 | | | |
| $\frac{39-6}{42-6}*100=92\%$ | | | |

**S.R.A.**^6^

Domain 1 (Scope and Purpose)

|  | Item 1 | Item 2 | Item 3 | Total |
| --- | --- | --- | --- | --- |
| Appraiser 1 (KSi) | 6 | 6 | 7 | 19 |
| Appraiser 2 (KSv) | 7 | 7 | 7 | 21 |
| Appraiser 3 (AR) | 7 | 7 | 7 | 21 |
| Total | 20 | 20 | 21 | 61 |
| Maximum possible score = 7 (strongly agree) x 3 (items) x 3 (appraisers) = 63  Minimum possible score = 1 (strongly disagree) x 3 (items) x 3 (appraisers) = 9 | | | | |
| $\frac{61-9}{63-9}*100=96 \%$ | | | | |

Domain 2 (Stakeholder Involvement)

|  | Item 4 | Item 5 | Item 6 | Total |
| --- | --- | --- | --- | --- |
| Appraiser 1 (KSi) | 6 | 5 | 7 | 18 |
| Appraiser 2 (KSv) | 6 | 5 | 7 | 18 |
| Appraiser 3 (AR) | 7 | 7 | 7 | 21 |
| Total | 19 | 17 | 21 | 57 |
| Maximum possible score = 7 (strongly agree) x 3 (items) x 3 (appraisers) = 63  Minimum possible score = 1 (strongly disagree) x 3 (items) x 3 (appraisers) = 9 | | | | |
| $\frac{57-9}{63-9}*100=89 \%$ | | | | |

Domain 3 (Rigour of Development)

|  | Item 7 | Item 8 | Item 9 | Item 10 | Item 11 | Item 12 | Item 13 | Item 14 | Total |
| --- | --- | --- | --- | --- | --- | --- | --- | --- | --- |
| Appraiser 1 (KSi) | 7 | 7 | 7 | 7 | 6 | 7 | 3 | 3 | 47 |
| Appraiser 2 (KSv) | 7 | 7 | 7 | 6 | 7 | 7 | 2 | 2 | 45 |
| Appraiser 3 (AR) | 7 | 7 | 7 | 7 | 7 | 7 | 3 | 4 | 49 |
| Total | 21 | 21 | 21 | 20 | 20 | 21 | 8 | 9 | 141 |
| Maximum possible score = 7 (strongly agree) x 8 (items) x 3 (appraisers) = 168  Minimum possible score = 1 (strongly disagree) x 8 (items) x 3 (appraisers) = 24 | | | | | | | | | |
| $\frac{141-24}{168-24}*100=81 \%$ | | | | | | | | | |

Domain 4 (Clarity of Presentation)

|  | Item 15 | Item 16 | Item 17 | Total |
| --- | --- | --- | --- | --- |
| Appraiser 1 (KSi) | 6 | 6 | 6 | 18 |
| Appraiser 2 (KSv) | 7 | 7 | 6 | 20 |
| Appraiser 3 (AR) | 7 | 7 | 7 | 21 |
| Total | 20 | 20 | 19 | 59 |
| Maximum possible score = 7 (strongly agree) x 3 (items) x 3 (appraisers) = 63  Minimum possible score = 1 (strongly disagree) x 3 (items) x 3 (appraisers) = 9 | | | | |
| $\frac{59-9}{63-9}*100=93 \%$ | | | | |

Domain 5 (Applicability)

|  | Item 18 | Item 19 | Item 20 | Item 21 | Total |
| --- | --- | --- | --- | --- | --- |
| Appraiser 1 (KSi) | 6 | 6 | 6 | 7 | 25 |
| Appraiser 2 (KSv) | 5 | 7 | 7 | 6 | 25 |
| Appraiser 3 (AR) | 5 | 4 | 7 | 6 | 22 |
| Total | 16 | 17 | 20 | 19 | 72 |
| Maximum possible score = 7 (strongly agree) x 4 (items) x 3 (appraisers) = 84  Minimum possible score = 1 (strongly disagree) x 4 (items) x 3 (appraisers) = 12 | | | | | |
| $\frac{72-12}{84-12}*100=83 \%$ | | | | | |

Domain 6 (Editorial Independence)

|  | Item 22 | Item 23 | Total |
| --- | --- | --- | --- |
| Appraiser 1 (KSi) | 1 | 7 | 8 |
| Appraiser 2 (KSv) | 1 | 7 | 8 |
| Appraiser 3 (AR) | 1 | 7 | 8 |
| Total | 3 | 21 | 24 |
| Maximum possible score = 7 (strongly agree) x 2 (items) x 3 (appraisers) = 42  Minimum possible score = 1 (strongly disagree) x 2 (items) x 3 (appraisers) = 6 | | | |
| $\frac{24-6}{42-6}*100=50 \%$ | | | |

**T.I.A.**^7^

Domain 1 (Scope and Purpose)

|  | Item 1 | Item 2 | Item 3 | Total |
| --- | --- | --- | --- | --- |
| Appraiser 1 (KSi) | 6 | 5 | 7 | 18 |
| Appraiser 2 (KSv) | 6 | 4 | 7 | 17 |
| Appraiser 3 (AR) | 7 | 4 | 7 | 18 |
| Total | 19 | 13 | 21 | 53 |
| Maximum possible score = 7 (strongly agree) x 3 (items) x 3 (appraisers) = 63  Minimum possible score = 1 (strongly disagree) x 3 (items) x 3 (appraisers) = 9 | | | | |
| $\frac{53-9}{63-9}*100=81 \%$ | | | | |

Domain 2 (Stakeholder Involvement)

|  | Item 4 | Item 5 | Item 6 | Total |
| --- | --- | --- | --- | --- |
| Appraiser 1 (KSi) | 5 | 5 | 7 | 17 |
| Appraiser 2 (KSv) | 4 | 5 | 7 | 16 |
| Appraiser 3 (AR) | 7 | 7 | 7 | 21 |
| Total | 16 | 17 | 21 | 54 |
| Maximum possible score = 7 (strongly agree) x 3 (items) x 3 (appraisers) = 63  Minimum possible score = 1 (strongly disagree) x 3 (items) x 3 (appraisers) = 9 | | | | |
| $\frac{54-9}{63-9}*100=83 \%$ | | | | |

Domain 3 (Rigour of Development)

|  | Item 7 | Item 8 | Item 9 | Item 10 | Item 11 | Item 12 | Item 13 | Item 14 | Total |
| --- | --- | --- | --- | --- | --- | --- | --- | --- | --- |
| Appraiser 1 (KSi) | 1 | 2 | 3 | 2 | 4 | 3 | 2 | 3 | 20 |
| Appraiser 2 (KSv) | 1 | 2 | 1 | 2 | 3 | 2 | 1 | 2 | 14 |
| Appraiser 3 (AR) | 2 | 3 | 3 | 4 | 3 | 4 | 2 | 5 | 26 |
| Total | 4 | 7 | 7 | 8 | 10 | 9 | 5 | 10 | 60 |
| Maximum possible score = 7 (strongly agree) x 8 (items) x 3 (appraisers) = 168  Minimum possible score = 1 (strongly disagree) x 8 (items) x 3 (appraisers) = 24 | | | | | | | | | |
| $\frac{60-24}{168-24}*100=25 \%$ | | | | | | | | | |

Domain 4 (Clarity of Presentation)

|  | Item 15 | Item 16 | Item 17 | Total |
| --- | --- | --- | --- | --- |
| Appraiser 1 (KSi) | 5 | 5 | 6 | 16 |
| Appraiser 2 (KSv) | 6 | 3 | 6 | 15 |
| Appraiser 3 (AR) | 7 | 7 | 7 | 21 |
| Total | 18 | 15 | 19 | 52 |
| Maximum possible score = 7 (strongly agree) x 3 (items) x 3 (appraisers) = 63  Minimum possible score = 1 (strongly disagree) x 3 (items) x 3 (appraisers) = 9 | | | | |
| $\frac{52-9}{63-9}*100=80 \%$ | | | | |

Domain 5 (Applicability)

|  | Item 18 | Item 19 | Item 20 | Item 21 | Total |
| --- | --- | --- | --- | --- | --- |
| Appraiser 1 (KSi) | 3 | 4 | 7 | 5 | 19 |
| Appraiser 2 (KSv) | 2 | 3 | 7 | 4 | 16 |
| Appraiser 3 (AR) | 4 | 4 | 7 | 6 | 21 |
| Total | 9 | 11 | 21 | 15 | 56 |
| Maximum possible score = 7 (strongly agree) x 4 (items) x 3 (appraisers) = 84  Minimum possible score = 1 (strongly disagree) x 4 (items) x 3 (appraisers) = 12 | | | | | |
| $\frac{56-12}{84-12}*100=61 \%$ | | | | | |

Domain 6 (Editorial Independence)

|  | Item 22 | Item 23 | Total |
| --- | --- | --- | --- |
| Appraiser 1 (KSi) | 3 | 2 | 5 |
| Appraiser 2 (KSv) | 3 | 1 | 4 |
| Appraiser 3 (AR) | 1 | 4 | 5 |
| Total | 7 | 7 | 14 |
| Maximum possible score = 7 (strongly agree) x 2 (items) x 3 (appraisers) = 42  Minimum possible score = 1 (strongly disagree) x 2 (items) x 3 (appraisers) = 6 | | | |
| $\frac{14-6}{42-6}*100=22 \%$ | | | |

**N.G.D.**^8^

Domain 1 (Scope and Purpose)

|  | Item 1 | Item 2 | Item 3 | Total |
| --- | --- | --- | --- | --- |
| Appraiser 1 (KSi) | 5 | 2 | 5 | 12 |
| Appraiser 2 (KSv) | 4 | 2 | 6 | 12 |
| Appraiser 3 (AR) | 3 | 2 | 4 | 9 |
| Total | 12 | 6 | 15 | 33 |
| Maximum possible score = 7 (strongly agree) x 3 (items) x 3 (appraisers) = 63  Minimum possible score = 1 (strongly disagree) x 3 (items) x 3 (appraisers) = 9 | | | | |
| $\frac{33-9}{63-9}*100=44 \%$ | | | | |

Domain 2 (Stakeholder Involvement)

|  | Item 4 | Item 5 | Item 6 | Total |
| --- | --- | --- | --- | --- |
| Appraiser 1 (KSi) | 1 | 1 | 2 | 4 |
| Appraiser 2 (KSv) | 1 | 1 | 1 | 3 |
| Appraiser 3 (AR) | 1 | 1 | 2 | 4 |
| Total | 3 | 3 | 5 | 11 |
| Maximum possible score = 7 (strongly agree) x 3 (items) x 3 (appraisers) = 63  Minimum possible score = 1 (strongly disagree) x 3 (items) x 3 (appraisers) = 9 | | | | |
| $\frac{11-9}{63-9}*100=4 \%$ | | | | |

Domain 3 (Rigour of Development)

|  | Item 7 | Item 8 | Item 9 | Item 10 | Item 11 | Item 12 | Item 13 | Item 14 | Total |
| --- | --- | --- | --- | --- | --- | --- | --- | --- | --- |
| Appraiser 1 (KSi) | 5 | 2 | 1 | 3 | 3 | 2 | 1 | 1 | 18 |
| Appraiser 2 (KSv) | 6 | 3 | 1 | 2 | 2 | 2 | 1 | 1 | 18 |
| Appraiser 3 (AR) | 4 | 2 | 1 | 2 | 4 | 2 | 1 | 1 | 17 |
| Total | 15 | 7 | 3 | 7 | 9 | 6 | 3 | 3 | 53 |
| Maximum possible score = 7 (strongly agree) x 8 (items) x 3 (appraisers) = 168  Minimum possible score = 1 (strongly disagree) x 8 (items) x 3 (appraisers) = 24 | | | | | | | | | |
| $\frac{53-24}{168-24}*100=20 \%$ | | | | | | | | | |

Domain 4 (Clarity of Presentation)

|  | Item 15 | Item 16 | Item 17 | Total |
| --- | --- | --- | --- | --- |
| Appraiser 1 (KSi) | 6 | 6 | 4 | 16 |
| Appraiser 2 (KSv) | 5 | 6 | 6 | 17 |
| Appraiser 3 (AR) | 4 | 5 | 2 | 11 |
| Total | 15 | 17 | 12 | 44 |
| Maximum possible score = 7 (strongly agree) x 3 (items) x 3 (appraisers) = 63  Minimum possible score = 1 (strongly disagree) x 3 (items) x 3 (appraisers) = 9 | | | | |
| $\frac{44-9}{63-9}*100=65 \%$ | | | | |

Domain 5 (Applicability)

|  | Item 18 | Item 19 | Item 20 | Item 21 | Total |
| --- | --- | --- | --- | --- | --- |
| Appraiser 1 (KSi) | 1 | 2 | 1 | 1 | 5 |
| Appraiser 2 (KSv) | 1 | 3 | 1 | 1 | 6 |
| Appraiser 3 (AR) | 1 | 3 | 1 | 1 | 6 |
| Total | 3 | 8 | 3 | 3 | 17 |
| Maximum possible score = 7 (strongly agree) x 4 (items) x 3 (appraisers) = 84  Minimum possible score = 1 (strongly disagree) x 4 (items) x 3 (appraisers) = 12 | | | | | |
| $\frac{17-12}{84-12}*100=7 \%$ | | | | | |

Domain 6 (Editorial Independence)

|  | Item 22 | Item 23 | Total |
| --- | --- | --- | --- |
| Appraiser 1 (KSi) | 7 | 3 | 10 |
| Appraiser 2 (KSv) | 7 | 2 | 9 |
| Appraiser 3 (AR) | 7 | 2 | 9 |
| Total | 21 | 7 | 28 |
| Maximum possible score = 7 (strongly agree) x 2 (items) x 3 (appraisers) = 42  Minimum possible score = 1 (strongly disagree) x 2 (items) x 3 (appraisers) = 6 | | | |
| $\frac{28-6}{42-6}*100=61 \%$ | | | |

**B.R.1.**^9^

Domain 1 (Scope and Purpose)

|  | Item 1 | Item 2 | Item 3 | Total |
| --- | --- | --- | --- | --- |
| Appraiser 1 (KSi) | 5 | 1 | 6 | 12 |
| Appraiser 2 (KSv) | 5 | 2 | 7 | 14 |
| Appraiser 3 (AR) | 6 | 1 | 5 | 12 |
| Total | 16 | 4 | 18 | 38 |
| Maximum possible score = 7 (strongly agree) x 3 (items) x 3 (appraisers) = 63  Minimum possible score = 1 (strongly disagree) x 3 (items) x 3 (appraisers) = 9 | | | | |
| $\frac{38-9}{63-9}*100=54 \%$ | | | | |

Domain 2 (Stakeholder Involvement)

|  | Item 4 | Item 5 | Item 6 | Total |
| --- | --- | --- | --- | --- |
| Appraiser 1 (KSi) | 1 | 1 | 6 | 8 |
| Appraiser 2 (KSv) | 1 | 1 | 7 | 9 |
| Appraiser 3 (AR) | 3 | 1 | 7 | 11 |
| Total | 5 | 3 | 20 | 28 |
| Maximum possible score = 7 (strongly agree) x 3 (items) x 3 (appraisers) = 63  Minimum possible score = 1 (strongly disagree) x 3 (items) x 3 (appraisers) = 9 | | | | |
| $\frac{28-9}{63-9}*100=35 \%$ | | | | |

Domain 3 (Rigour of Development)

|  | Item 7 | Item 8 | Item 9 | Item 10 | Item 11 | Item 12 | Item 13 | Item 14 | Total |
| --- | --- | --- | --- | --- | --- | --- | --- | --- | --- |
| Appraiser 1 (KSi) | 2 | 1 | 2 | 3 | 2 | 2 | 1 | 2 | 15 |
| Appraiser 2 (KSv) | 1 | 2 | 2 | 2 | 2 | 2 | 1 | 1 | 13 |
| Appraiser 3 (AR) | 1 | 1 | 1 | 2 | 4 | 2 | 1 | 1 | 13 |
| Total | 4 | 4 | 5 | 7 | 8 | 6 | 3 | 4 | 41 |
| Maximum possible score = 7 (strongly agree) x 8 (items) x 3 (appraisers) = 168  Minimum possible score = 1 (strongly disagree) x 8 (items) x 3 (appraisers) = 24 | | | | | | | | | |
| $\frac{41-24}{168-24}*100=12 \%$ | | | | | | | | | |

Domain 4 (Clarity of Presentation)

|  | Item 15 | Item 16 | Item 17 | Total |
| --- | --- | --- | --- | --- |
| Appraiser 1 (KSi) | 4 | 2 | 4 | 10 |
| Appraiser 2 (KSv) | 5 | 1 | 6 | 12 |
| Appraiser 3 (AR) | 6 | 4 | 5 | 15 |
| Total | 15 | 7 | 15 | 37 |
| Maximum possible score = 7 (strongly agree) x 3 (items) x 3 (appraisers) = 63  Minimum possible score = 1 (strongly disagree) x 3 (items) x 3 (appraisers) = 9 | | | | |
| $\frac{37-9}{63-9}*100=52 \%$ | | | | |

Domain 5 (Applicability)

|  | Item 18 | Item 19 | Item 20 | Item 21 | Total |
| --- | --- | --- | --- | --- | --- |
| Appraiser 1 (KSi) | 1 | 1 | 1 | 1 | 4 |
| Appraiser 2 (KSv) | 1 | 1 | 1 | 1 | 4 |
| Appraiser 3 (AR) | 3 | 2 | 1 | 1 | 7 |
| Total | 5 | 4 | 3 | 3 | 15 |
| Maximum possible score = 7 (strongly agree) x 4 (items) x 3 (appraisers) = 84  Minimum possible score = 1 (strongly disagree) x 4 (items) x 3 (appraisers) = 12 | | | | | |
| $\frac{15-12}{84-12}*100=4 \%$ | | | | | |

Domain 6 (Editorial Independence)

|  | Item 22 | Item 23 | Total |
| --- | --- | --- | --- |
| Appraiser 1 (KSi) | 1 | 3 | 4 |
| Appraiser 2 (KSv) | 1 | 2 | 3 |
| Appraiser 3 (AR) | 1 | 2 | 3 |
| Total | 3 | 7 | 10 |
| Maximum possible score = 7 (strongly agree) x 2 (items) x 3 (appraisers) = 42  Minimum possible score = 1 (strongly disagree) x 2 (items) x 3 (appraisers) = 6 | | | |
| $\frac{10-6}{42-6}*100=11 \%$ | | | |

**B.R.2.**^10^

Domain 1 (Scope and Purpose)

|  | Item 1 | Item 2 | Item 3 | Total |
| --- | --- | --- | --- | --- |
| Appraiser 1 (KSi) | 4 | 1 | 3 | 8 |
| Appraiser 2 (KSv) | 5 | 2 | 4 | 11 |
| Appraiser 3 (AR) | 6 | 1 | 4 | 11 |
| Total | 15 | 4 | 11 | 30 |
| Maximum possible score = 7 (strongly agree) x 3 (items) x 3 (appraisers) = 63  Minimum possible score = 1 (strongly disagree) x 3 (items) x 3 (appraisers) = 9 | | | | |
| $\frac{30-9}{63-9}*100=39 \%$ | | | | |

Domain 2 (Stakeholder Involvement)

|  | Item 4 | Item 5 | Item 6 | Total |
| --- | --- | --- | --- | --- |
| Appraiser 1 (KSi) | 1 | 1 | 4 | 6 |
| Appraiser 2 (KSv) | 1 | 1 | 5 | 7 |
| Appraiser 3 (AR) | 3 | 1 | 5 | 9 |
| Total | 5 | 3 | 14 | 22 |
| Maximum possible score = 7 (strongly agree) x 3 (items) x 3 (appraisers) = 63  Minimum possible score = 1 (strongly disagree) x 3 (items) x 3 (appraisers) = 9 | | | | |
| $\frac{22-9}{63-9}*100=24 \%$ | | | | |

Domain 3 (Rigour of Development)

|  | Item 7 | Item 8 | Item 9 | Item 10 | Item 11 | Item 12 | Item 13 | Item 14 | Total |
| --- | --- | --- | --- | --- | --- | --- | --- | --- | --- |
| Appraiser 1 (KSi) | 1 | 1 | 2 | 3 | 2 | 2 | 1 | 1 | 13 |
| Appraiser 2 (KSv) | 1 | 2 | 2 | 2 | 3 | 2 | 1 | 1 | 14 |
| Appraiser 3 (AR) | 1 | 1 | 2 | 2 | 4 | 2 | 1 | 1 | 14 |
| Total | 3 | 4 | 6 | 7 | 9 | 6 | 3 | 3 | 41 |
| Maximum possible score = 7 (strongly agree) x 8 (items) x 3 (appraisers) = 168  Minimum possible score = 1 (strongly disagree) x 8 (items) x 3 (appraisers) = 24 | | | | | | | | | |
| $\frac{41-24}{168-24}*100=12 \%$ | | | | | | | | | |

Domain 4 (Clarity of Presentation)

|  | Item 15 | Item 16 | Item 17 | Total |
| --- | --- | --- | --- | --- |
| Appraiser 1 (KSi) | 3 | 2 | 4 | 9 |
| Appraiser 2 (KSv) | 4 | 1 | 5 | 10 |
| Appraiser 3 (AR) | 3 | 4 | 5 | 12 |
| Total | 10 | 7 | 14 | 31 |
| Maximum possible score = 7 (strongly agree) x 3 (items) x 3 (appraisers) = 63  Minimum possible score = 1 (strongly disagree) x 3 (items) x 3 (appraisers) = 9 | | | | |
| $\frac{31-9}{63-9}*100=41 \%$ | | | | |

Domain 5 (Applicability)

|  | Item 18 | Item 19 | Item 20 | Item 21 | Total |
| --- | --- | --- | --- | --- | --- |
| Appraiser 1 (KSi) | 1 | 1 | 1 | 1 | 4 |
| Appraiser 2 (KSv) | 1 | 1 | 1 | 1 | 4 |
| Appraiser 3 (AR) | 1 | 1 | 1 | 1 | 4 |
| Total | 3 | 3 | 3 | 3 | 12 |
| Maximum possible score = 7 (strongly agree) x 4 (items) x 3 (appraisers) = 84  Minimum possible score = 1 (strongly disagree) x 4 (items) x 3 (appraisers) = 12 | | | | | |
| $\frac{12-12}{84-12}*100=0 \%$ | | | | | |

Domain 6 (Editorial Independence)

|  | Item 22 | Item 23 | Total |
| --- | --- | --- | --- |
| Appraiser 1 (KSi) | 1 | 3 | 4 |
| Appraiser 2 (KSv) | 1 | 2 | 3 |
| Appraiser 3 (AR) | 1 | 3 | 4 |
| Total | 3 | 8 | 11 |
| Maximum possible score = 7 (strongly agree) x 2 (items) x 3 (appraisers) = 42  Minimum possible score = 1 (strongly disagree) x 2 (items) x 3 (appraisers) = 6 | | | |
| $\frac{11-6}{42-6}*100=14 \%$ | | | |

**C.S.M.**^11^

Domain 1 (Scope and Purpose)

|  | Item 1 | Item 2 | Item 3 | Total |
| --- | --- | --- | --- | --- |
| Appraiser 1 (KSi) | 7 | 2 | 5 | 14 |
| Appraiser 2 (KSv) | 7 | 3 | 6 | 16 |
| Appraiser 3 (AR) | 6 | 1 | 4 | 11 |
| Total | 20 | 6 | 15 | 41 |
| Maximum possible score = 7 (strongly agree) x 3 (items) x 3 (appraisers) = 63  Minimum possible score = 1 (strongly disagree) x 3 (items) x 3 (appraisers) = 9 | | | | |
| $\frac{41-9}{63-9}*100=59 \%$ | | | | |

Domain 2 (Stakeholder Involvement)

|  | Item 4 | Item 5 | Item 6 | Total |
| --- | --- | --- | --- | --- |
| Appraiser 1 (KSi) | 5 | 6 | 5 | 16 |
| Appraiser 2 (KSv) | 5 | 7 | 7 | 19 |
| Appraiser 3 (AR) | 4 | 6 | 5 | 15 |
| Total | 14 | 19 | 17 | 50 |
| Maximum possible score = 7 (strongly agree) x 3 (items) x 3 (appraisers) = 63  Minimum possible score = 1 (strongly disagree) x 3 (items) x 3 (appraisers) = 9 | | | | |
| $\frac{50-9}{63-9}*100=76 \%$ | | | | |

Domain 3 (Rigour of Development)

|  | Item 7 | Item 8 | Item 9 | Item 10 | Item 11 | Item 12 | Item 13 | Item 14 | Total |
| --- | --- | --- | --- | --- | --- | --- | --- | --- | --- |
| Appraiser 1 (KSi) | 5 | 4 | 4 | 6 | 6 | 4 | 4 | 4 | 37 |
| Appraiser 2 (KSv) | 4 | 3 | 5 | 5 | 6 | 6 | 5 | 5 | 39 |
| Appraiser 3 (AR) | 5 | 5 | 5 | 6 | 5 | 6 | 3 | 4 | 39 |
| Total | 14 | 12 | 14 | 17 | 17 | 16 | 12 | 13 | 115 |
| Maximum possible score = 7 (strongly agree) x 8 (items) x 3 (appraisers) = 168  Minimum possible score = 1 (strongly disagree) x 8 (items) x 3 (appraisers) = 24 | | | | | | | | | |
| $\frac{115-24}{168-24}*100=63 \%$ | | | | | | | | | |

Domain 4 (Clarity of Presentation)

|  | Item 15 | Item 16 | Item 17 | Total |
| --- | --- | --- | --- | --- |
| Appraiser 1 (KSi) | 6 | 6 | 6 | 18 |
| Appraiser 2 (KSv) | 6 | 6 | 6 | 18 |
| Appraiser 3 (AR) | 4 | 6 | 6 | 16 |
| Total | 16 | 18 | 18 | 52 |
| Maximum possible score = 7 (strongly agree) x 3 (items) x 3 (appraisers) = 63  Minimum possible score = 1 (strongly disagree) x 3 (items) x 3 (appraisers) = 9 | | | | |
| $\frac{52-9}{63-9}*100=80 \%$ | | | | |

Domain 5 (Applicability)

|  | Item 18 | Item 19 | Item 20 | Item 21 | Total |
| --- | --- | --- | --- | --- | --- |
| Appraiser 1 (KSi) | 6 | 7 | 2 | 5 | 20 |
| Appraiser 2 (KSv) | 7 | 7 | 3 | 5 | 22 |
| Appraiser 3 (AR) | 6 | 7 | 3 | 5 | 21 |
| Total | 19 | 21 | 8 | 15 | 63 |
| Maximum possible score = 7 (strongly agree) x 4 (items) x 3 (appraisers) = 84  Minimum possible score = 1 (strongly disagree) x 4 (items) x 3 (appraisers) = 12 | | | | | |
| $\frac{63-12}{84-12}*100=71 \%$ | | | | | |

Domain 6 (Editorial Independence)

|  | Item 22 | Item 23 | Total |
| --- | --- | --- | --- |
| Appraiser 1 (KSi) | 7 | 7 | 14 |
| Appraiser 2 (KSv) | 7 | 7 | 14 |
| Appraiser 3 (AR) | 6 | 6 | 12 |
| Total | 20 | 20 | 40 |
| Maximum possible score = 7 (strongly agree) x 2 (items) x 3 (appraisers) = 42  Minimum possible score = 1 (strongly disagree) x 2 (items) x 3 (appraisers) = 6 | | | |
| $\frac{40-6}{42-6}*100=94 \%$ | | | |

**C.R.R.**^12^

Domain 1 (Scope and Purpose)

|  | Item 1 | Item 2 | Item 3 | Total |
| --- | --- | --- | --- | --- |
| Appraiser 1 (KSi) | 7 | 2 | 5 | 14 |
| Appraiser 2 (KSv) | 7 | 2 | 6 | 15 |
| Appraiser 3 (AR) | 6 | 1 | 6 | 13 |
| Total | 20 | 5 | 17 | 42 |
| Maximum possible score = 7 (strongly agree) x 3 (items) x 3 (appraisers) = 63  Minimum possible score = 1 (strongly disagree) x 3 (items) x 3 (appraisers) = 9 | | | | |
| $\frac{42-9}{63-9}*100=61 \%$ | | | | |

Domain 2 (Stakeholder Involvement)

|  | Item 4 | Item 5 | Item 6 | Total |
| --- | --- | --- | --- | --- |
| Appraiser 1 (KSi) | 7 | 6 | 6 | 19 |
| Appraiser 2 (KSv) | 7 | 7 | 7 | 21 |
| Appraiser 3 (AR) | 6 | 6 | 5 | 17 |
| Total | 20 | 19 | 18 | 57 |
| Maximum possible score = 7 (strongly agree) x 3 (items) x 3 (appraisers) = 63  Minimum possible score = 1 (strongly disagree) x 3 (items) x 3 (appraisers) = 9 | | | | |
| $\frac{57-9}{63-9}*100=89 \%$ | | | | |

Domain 3 (Rigour of Development)

|  | Item 7 | Item 8 | Item 9 | Item 10 | Item 11 | Item 12 | Item 13 | Item 14 | Total |
| --- | --- | --- | --- | --- | --- | --- | --- | --- | --- |
| Appraiser 1 (KSi) | 6 | 4 | 4 | 3 | 6 | 4 | 4 | 3 | 34 |
| Appraiser 2 (KSv) | 4 | 3 | 4 | 4 | 6 | 6 | 6 | 3 | 36 |
| Appraiser 3 (AR) | 4 | 5 | 5 | 3 | 6 | 6 | 4 | 2 | 35 |
| Total | 14 | 12 | 13 | 10 | 18 | 16 | 14 | 8 | 105 |
| Maximum possible score = 7 (strongly agree) x 8 (items) x 3 (appraisers) = 168  Minimum possible score = 1 (strongly disagree) x 8 (items) x 3 (appraisers) = 24 | | | | | | | | | |
| $\frac{105-24}{168-24}*100=56 \%$ | | | | | | | | | |

Domain 4 (Clarity of Presentation)

|  | Item 15 | Item 16 | Item 17 | Total |
| --- | --- | --- | --- | --- |
| Appraiser 1 (KSi) | 5 | 7 | 6 | 18 |
| Appraiser 2 (KSv) | 6 | 7 | 6 | 19 |
| Appraiser 3 (AR) | 5 | 7 | 6 | 18 |
| Total | 16 | 21 | 18 | 55 |
| Maximum possible score = 7 (strongly agree) x 3 (items) x 3 (appraisers) = 63  Minimum possible score = 1 (strongly disagree) x 3 (items) x 3 (appraisers) = 9 | | | | |
| $\frac{55-9}{63-9}*100=85 \%$ | | | | |

Domain 5 (Applicability)

|  | Item 18 | Item 19 | Item 20 | Item 21 | Total |
| --- | --- | --- | --- | --- | --- |
| Appraiser 1 (KSi) | 6 | 7 | 2 | 6 | 21 |
| Appraiser 2 (KSv) | 7 | 7 | 3 | 6 | 23 |
| Appraiser 3 (AR) | 5 | 7 | 1 | 6 | 19 |
| Total | 18 | 21 | 6 | 18 | 63 |
| Maximum possible score = 7 (strongly agree) x 4 (items) x 3 (appraisers) = 84  Minimum possible score = 1 (strongly disagree) x 4 (items) x 3 (appraisers) = 12 | | | | | |
| $\frac{63-12}{84-12}*100=71 \%$ | | | | | |

Domain 6 (Editorial Independence)

|  | Item 22 | Item 23 | Total |
| --- | --- | --- | --- |
| Appraiser 1 (KSi) | 7 | 7 | 14 |
| Appraiser 2 (KSv) | 7 | 7 | 14 |
| Appraiser 3 (AR) | 6 | 6 | 12 |
| Total | 20 | 20 | 40 |
| Maximum possible score = 7 (strongly agree) x 2 (items) x 3 (appraisers) = 42  Minimum possible score = 1 (strongly disagree) x 2 (items) x 3 (appraisers) = 6 | | | |
| $\frac{40-6}{42-6}*100=94 \%$ | | | |

**E.S.P.**^13^

Domain 1 (Scope and Purpose)

|  | Item 1 | Item 2 | Item 3 | Total |
| --- | --- | --- | --- | --- |
| Appraiser 1 (KSi) | 6 | 6 | 6 | 18 |
| Appraiser 2 (KSv) | 6 | 5 | 7 | 18 |
| Appraiser 3 (AR) | 6 | 6 | 4 | 16 |
| Total | 18 | 17 | 17 | 52 |
| Maximum possible score = 7 (strongly agree) x 3 (items) x 3 (appraisers) = 63  Minimum possible score = 1 (strongly disagree) x 3 (items) x 3 (appraisers) = 9 | | | | |
| $\frac{52-9}{63-9}*100=80 \%$ | | | | |

Domain 2 (Stakeholder Involvement)

|  | Item 4 | Item 5 | Item 6 | Total |
| --- | --- | --- | --- | --- |
| Appraiser 1 (KSi) | 3 | 1 | 6 | 10 |
| Appraiser 2 (KSv) | 4 | 1 | 7 | 12 |
| Appraiser 3 (AR) | 4 | 1 | 6 | 11 |
| Total | 11 | 3 | 19 | 33 |
| Maximum possible score = 7 (strongly agree) x 3 (items) x 3 (appraisers) = 63  Minimum possible score = 1 (strongly disagree) x 3 (items) x 3 (appraisers) = 9 | | | | |
| $\frac{533-9}{63-9}*100=44 \%$ | | | | |

Domain 3 (Rigour of Development)

|  | Item 7 | Item 8 | Item 9 | Item 10 | Item 11 | Item 12 | Item 13 | Item 14 | Total |
| --- | --- | --- | --- | --- | --- | --- | --- | --- | --- |
| Appraiser 1 (KSi) | 6 | 4 | 6 | 6 | 4 | 6 | 1 | 3 | 36 |
| Appraiser 2 (KSv) | 5 | 4 | 7 | 7 | 5 | 7 | 1 | 4 | 40 |
| Appraiser 3 (AR) | 5 | 2 | 6 | 6 | 5 | 7 | 1 | 3 | 35 |
| Total | 16 | 10 | 19 | 19 | 14 | 20 | 3 | 10 | 111 |
| Maximum possible score = 7 (strongly agree) x 8 (items) x 3 (appraisers) = 168  Minimum possible score = 1 (strongly disagree) x 8 (items) x 3 (appraisers) = 24 | | | | | | | | | |
| $\frac{111-24}{168-24}*100=60 \%$ | | | | | | | | | |

Domain 4 (Clarity of Presentation)

|  | Item 15 | Item 16 | Item 17 | Total |
| --- | --- | --- | --- | --- |
| Appraiser 1 (KSi) | 5 | 4 | 4 | 13 |
| Appraiser 2 (KSv) | 5 | 5 | 4 | 14 |
| Appraiser 3 (AR) | 5 | 6 | 4 | 15 |
| Total | 15 | 15 | 12 | 42 |
| Maximum possible score = 7 (strongly agree) x 3 (items) x 3 (appraisers) = 63  Minimum possible score = 1 (strongly disagree) x 3 (items) x 3 (appraisers) = 9 | | | | |
| $\frac{42-9}{63-9}*100=61 \%$ | | | | |

Domain 5 (Applicability)

|  | Item 18 | Item 19 | Item 20 | Item 21 | Total |
| --- | --- | --- | --- | --- | --- |
| Appraiser 1 (KSi) | 1 | 2 | 2 | 1 | 6 |
| Appraiser 2 (KSv) | 1 | 1 | 1 | 1 | 4 |
| Appraiser 3 (AR) | 2 | 2 | 1 | 1 | 6 |
| Total | 4 | 5 | 4 | 3 | 16 |
| Maximum possible score = 7 (strongly agree) x 4 (items) x 3 (appraisers) = 84  Minimum possible score = 1 (strongly disagree) x 4 (items) x 3 (appraisers) = 12 | | | | | |
| $\frac{16-12}{84-12}*100=6 \%$ | | | | | |

Domain 6 (Editorial Independence)

|  | Item 22 | Item 23 | Total |
| --- | --- | --- | --- |
| Appraiser 1 (KSi) | 2 | 4 | 6 |
| Appraiser 2 (KSv) | 1 | 5 | 6 |
| Appraiser 3 (AR) | 1 | 5 | 6 |
| Total | 4 | 14 | 18 |
| Maximum possible score = 7 (strongly agree) x 2 (items) x 3 (appraisers) = 42  Minimum possible score = 1 (strongly disagree) x 2 (items) x 3 (appraisers) = 6 | | | |
| $\frac{18-6}{42-6}*100=33 \%$ | | | |

**References**

1. Powers WJ, Rabinstein AA, Ackerson T, et al. Guidelines for the Early Management of Patients With Acute Ischemic Stroke: 2019 Update to the 2018 Guidelines for the Early Management of Acute Ischemic Stroke: A Guideline for Healthcare Professionals From the American Heart Association/American Stroke Association. *Stroke*. 2019;50(12):e344-e418. doi:10.1161/STR.0000000000000211

2. Greenberg SM, Ziai WC, Cordonnier C, et al. 2022 Guideline for the Management of Patients With Spontaneous Intracerebral Hemorrhage: A Guideline From the American Heart Association/American Stroke Association. *Stroke*. 2022;53(7):e282-e361. doi:10.1161/STR.0000000000000407

3. Dziewas R, Michou E, Trapl-Grundschober M, et al. European Stroke Organisation and European Society for Swallowing Disorders guideline for the diagnosis and treatment of post-stroke dysphagia. *Eur Stroke J*. 2021;6(3):LXXXIX-CXV. doi:10.1177/23969873211039721

4. Stroke Foundation. Clinical Guidelines for Stroke Management. Available at https://informme.org.au/guidelines/living-clinical-guidelines-for-stroke-management. InformMe. Accessed October 16, 2024. https://informme.org.au/guidelines/living-clinical-guidelines-for-stroke-management

5. National Clinical Guideline for Stroke for the UK and Ireland. London: Intercollegiate Stroke Working Party; 2023 May 4. Available at: www.strokeguideline.org. National Clinical Guideline for Stroke. Accessed October 16, 2024. https://www.strokeguideline.org/

6. *Stroke Rehabilitation in Adults. London: National Institute for Health and Care Excellence (NICE); 2023 Oct 18. (NICE Clinical Guidelines, No. 236.) Available from: Https://Www.Ncbi.Nlm.Nih.Gov/Books/NBK598564/*. National Institute for Health and Care Excellence (NICE); 2023. Accessed October 16, 2024. http://www.ncbi.nlm.nih.gov/books/NBK598564/

7. *Stroke and Transient Ischaemic Attack in over 16s: Diagnosis and Initial Management. London: National Institute for Health and Care Excellence (NICE); 2022 Apr 13. (NICE Guideline, No. 128.) Available from: Https://Www.Ncbi.Nlm.Nih.Gov/Books/NBK542436/*. National Institute for Health and Care Excellence (NICE); 2022. Accessed October 16, 2024. http://www.ncbi.nlm.nih.gov/books/NBK542436/

8. Dziewas R, Allescher HD, Aroyo I, et al. Diagnosis and treatment of neurogenic dysphagia - S1 guideline of the German Society of Neurology. *Neurol Res Pract*. 2021;3(1):23. doi:10.1186/s42466-021-00122-3

9. Minelli C, Bazan R, Pedatella MTA, et al. Brazilian Academy of Neurology practice guidelines for stroke rehabilitation: part I. *Arq Neuropsiquiatr*. 2022;80:634-652. doi:10.1590/0004-282X-ANP-2021-0354

10. Minelli C, Luvizutto GJ, Cacho R de O, et al. Brazilian practice guidelines for stroke rehabilitation: Part II. *Arq Neuropsiquiatr*. 2022;80(7):741-758. doi:10.1055/s-0042-1757692

11. Heran M, Lindsay P, Gubitz G, et al. Canadian Stroke Best Practice Recommendations: Acute Stroke Management, 7th Edition Practice Guidelines Update, 2022. *Can J Neurol Sci J Can Sci Neurol*. 2024;51(1):1-31. doi:10.1017/cjn.2022.344

12. Teasell R, Salbach NM, Foley N, et al. Canadian Stroke Best Practice Recommendations: Rehabilitation, Recovery, and Community Participation following Stroke. Part One: Rehabilitation and Recovery Following Stroke; 6th Edition Update 2019. *Int J Stroke Off J Int Stroke Soc*. 2020;15(7):763-788. doi:10.1177/1747493019897843

13. R T, O A, E I, et al. ESPEN guideline on hospital nutrition. *Clin Nutr Edinb Scotl*. 2021;40(12). doi:10.1016/j.clnu.2021.09.039
