# Supplementary material for: Nutritional care in rehabilitation and acute care of stroke patients: a systematic review of clinical practice guidelines
Source: Front Stroke. 2025 Apr 10;4:1558019. doi: 10.3389/fstro.2025.1558019 (PMC12802607; doi:10.3389/fstro.2025.1558019)
Supplement: Supplementary file 5 [file Table_5.docx]

| **Supplementary Material Table S5: Quality Assessment (AGREE-II)** | | | | | | | |
| --- | --- | --- | --- | --- | --- | --- | --- |
| **Scaled domain score in %** | **Domain 1** | **Domain 2** | **Domain 3** | **Domain 4** | **Domain 5** | **Domain 6** | **T otal** |
| A.I.S.^18^ | **50** | **37** | **56** | **67** | **1** | **47** | **44** |
| I.C.H.^19^ | **63** | **48** | **58** | **80** | **10** | **86** | **54** |
| E.S.O.^15^ | **85** | **65** | **74** | **85** | **21** | **94** | **68** |
| S.F.M.^8^ | **100** | **87** | **88** | **98** | **68** | **72** | **86** |
| I.S.C.^16^ | **80** | **80** | **58** | **57** | **60** | **92** | **67** |
| S.R.A.^9^ | **96** | **89** | **81** | **93** | **83** | **50** | **83** |
| T.I.A.^17^ | **81** | **83** | **25** | **80** | **61** | **22** | **53** |
| N.G.D.^12^ | **44** | **4** | **20** | **65** | **7** | **61** | **28** |
| B.R.1.^13^ | **54** | **35** | **12** | **52** | **4** | **11** | **24** |
| B.R.2.^14^ | **39** | **24** | **12** | **41** | **0** | **14** | **19** |
| B.R.2.^14^ | **59** | **76** | **63** | **80** | **71** | **94** | **71** |
| C.S.M.^10^ | **61** | **89** | **56** | **85** | **71** | **94** | **71** |
| E.S.P.^7^ | **80** | **44** | **60** | **61** | **6** | **33** | **49** |
| **Mean [SD]** | **67.7 [20.2]** | **59.8 [29.2]** | **50.3 [26.6]** | **73.6 [17.3]** | **38.1 [33.2]** | **61.4 [32.4]** | **55.7 [22.7]** |
| Abbreviations: AGREE-II: appraisal of guidelines for research & evaluation II, SD: standard deviation | | | | | | | |
